# Subunits of the *Drosophila* Actin-Capping Protein Heterodimer Regulate Each Other at Multiple Levels

**DOI:** 10.1371/journal.pone.0096326

**Published:** 2014-05-02

**Authors:** Ana Rita Amândio, Pedro Gaspar, Jessica L. Whited, Florence Janody

**Affiliations:** Instituto Gulbenkian de Ciência, Oeiras, Portugal; MRC, University College of London, United Kingdom

## Abstract

The actin-Capping Protein heterodimer, composed of the α and β subunits, is a master F-actin regulator. In addition to its role in many cellular processes, Capping Protein acts as a main tumor suppressor module in *Drosophila* and in humans, in part, by restricting the activity of Yorkie/YAP/TAZ oncogenes. We aimed in this report to understand how both subunits regulate each other *in vivo*. We show that the levels and capping activities of both subunits must be tightly regulated to control F-actin levels and consequently growth of the *Drosophila* wing. Overexpressing *capping protein* α and β decreases both F-actin levels and tissue growth, while expressing forms of Capping Protein that have dominant negative effects on F-actin promote tissue growth. Both subunits regulate each other's protein levels. In addition, overexpressing one of the subunit in tissues knocked-down for the other increases the mRNA and protein levels of the subunit knocked-down and compensates for its loss. We propose that the ability of the α and β subunits to control each other's levels assures that a pool of functional heterodimer is produced in sufficient quantities to restrict the development of tumor but not in excess to sustain normal tissue growth.

## Introduction

The actin cytoskeleton controls numerous processes, including cell shape, mobility, division and intracellular transport. In normal cells, the actin cytoskeleton is tightly controlled to regulate these essential functions; however, it can be subverted by cancer cells and contributes to changes in cell growth, proliferation, stiffness, movement and invasiveness [1], [2]. Moreover, alterations in the activity or expression of actin-binding proteins (ABPs) *per se*, have been linked to cancer initiation and progression [2], [3], [4], [5], [6].

Among these actin regulators, the actin Capping Protein (CP) heterodimer, composed of an α and a β subunit, appears to act as a main tumor suppressor module [7], [8], [9], [10]. CP was named based on its ability to bind and cap actin filament barbed ends, inhibiting the addition and loss of actin monomers [11], [12], [13]. CP has homologs in nearly all eukaryotic cells, including vertebrates, invertebrates, plants, fungi, insects and protozoa [14]. *Drosophila* and organisms other than vertebrates have single genes encoding *capping protein α (cpa)* or *β (cpb)*. In contrast, vertebrates contain two genes expressed somatically that encode two α subunits (α1 and α2), and one single gene that produce two β isoforms (β1 and β2) through alternative splicing [15], [16], [17]. Although the amino acid sequences of the α and β subunits are not more similar to each other than they are to other ABPs, nor they share common sequences with other proteins, they have extremely similar secondary and tertiary structures [18]. When in complex, the heterodimer resembles a mushroom with the C-terminus of each subunit forming tentacles located on the top surface of the heterodimer [19], [20]. *In vitro* analyses of chicken and budding yeast CP revealed that deletions or point mutations in either the α or β tentacles do not affect protein stability but reduce the capping affinity, while a complete removal of both tentacles fully abrogates the actin-binding activity [12], [20]. Thus, CP appears to cap F-actin barbed ends via the independent interaction of both tentacles with actin. *In vivo*, a truncated form of *Drosophila cpa* deleted of the C-terminal 28 amino acids has no effect on F-actin when expressed alone but promotes F-actin accumulation when co-expressed with full length *cpb* [21]. Similarly, a chicken β subunit containing a point mutation changing a conserved leucine to arginine at position 262, which caps actin poorly, disrupts the early steps in myofibrillogenesis of cultured myotubes and the sarcomere of mouse heart [22], [23], [24].

In yeast and *Drosophila*, removing either *cpa* or *cpb* induces F-actin accumulation and identical phenotypes [25], [26], [27]. In the fly, CP is required for proper differentiation of adult bristles, survival of the adult retina, determination of the oocyte and cortical integrity of nurse cells in the egg chamber [27], [28], [29], [30]. In addition, CP has a key role in restricting tissue growth. In the whole wing disc epithelium, CP-dependent F-actin regulation suppresses inappropriate tissue growth by inhibiting the activity of the Yorkie (Yki) oncogene, which mediates Hippo signalling activity [7], [9]. This function is conserved, as the α1 subunit is also required to limit the activity of the Yki orthologs YAP and TAZ in mammary epithelial cells [31]. In addition, in the distal *Drosophila* wing disc epithelium, CP prevents JNK-mediated apoptosis or proliferation and counteracts the oncogenic ability of Src [8], [21], [32]. Furthermore, underexpression of the human α1 subunit correlates with cancer-related death and causes a significant increase in gastric cancer cell migration and invasion *in vitro*, whereas its overexpression has the opposite effect [10].

We aimed in this report to understand how both subunits regulate each other *in vivo* to control F-actin levels and tissue growth. We show that Cpa and Cpb stabilize each other's protein levels and can stimulate the production of each other's mRNA when the level of one of the subunit is reduced. Because overexpressing *CP* decreases F-actin levels and tissue growth, while expressing forms of CP mutated in their actin-binding domains has opposite effects, we propose that by regulating each other, Cpa and Cpb assure that a pool of functional CP heterodimer is produced in sufficient quantities to restrict tissue growth and therein prevent tumor development but not in excess to sustain proper tissue growth.

## Materials and Methods

### Molecular Biology

To generate *UAS-cpb^L262R^*, site-directed mutagenesis was performed on the plasmid UAS-*cpb*, using the QuikChange kit (Stratagene, # 200519). The mutated plasmid was confirmed by sequencing and transgenic flies were generated by standard methods.

### Fly strains and genetics

Fly stocks used were *sd*-Gal4 [33]; *nub*-Gal4 [34]; *hh*-Gal4 (a gift from T. Tabata); *da*-Gal4 [35]; UAS-*cpa-IR^C10^*, *UAS-cpa-IR^B4^* [7]; UAS-*HA-cpa^89E^*, UAS-*HA-cpa^ΔABD^* [21]; UAS-cpb7 [36]; UAS-*cpb-RI* ^45668^ (Vienna *Drosophila* Research Center, VDRC); *cpa^107E^* [25], *cpb^M143^* (FlyBase). To generate *cpa* mutant clones marked by the absence of GFP and expressing or not UAS-*HA-cpa^89E^* or UAS-*HA-cpa^ΔABD^* or UAS-*cpb^7^*, *w*; FRT42D, *cpa^69E^*/CyO or *w*; FRT42D, *cpa^107E^*/CyO; UAS-*HA-cpa^89E^*/Tm6β or *w*; FRT42D, *cpa^107E^*/CyO; UAS*-HA-cpa^ΔABD^*/Tm6β or *w*; FRT42D, *cpa^107E^*, UAS*-cpb^7^*/CyO males were crossed to *y, w,* FRT42D, *ubi*-GFP; *T155*-Gal4, UAS-*flp*/ST females. To generate *cpb* mutant clones marked by the absence of GFP, *w, y*; FRT40A, *cpb^M143^*/CyOy*^+^* males were crossed to *y, w, hs*FLP122; FRT40A, *ubi*-GFP females and the progeny was heat-shocked at first and second instar larvae. All crosses were maintained at 25°C and the progeny was dissected at end of third instar larvae.

### Antibody Generation

The rabbit anti-Cpa and rabbit anti-Cpb polyclonal antibodies were generated by Metabion International AG using full length Cpa or Cpb tagged with Histidine.

### Immunohistochemistry and quantification

We performed immunocytochemistry using the procedure described in Lee and Treisman [37]. Primary antibodies used were mouse anti-Arm (N2 7A1, Developmental Studies Hybridoma Bank (DSHB); 1∶10), rat anti-DE-Cad (1∶50, CAD2, DSHB), rabbit anti-Cpa (1∶200); rabbit anti-Cpb (1∶200); mouse anti-HA (Covance 11 MMS101P; 1∶1000) and rabbit anti-Caspase 3 (Cell Signalling #9661; 1∶50). Rhodamine conjugated phalloidin (Sigma) was used at a concentration of 0.3 µM. Secondary antibodies were from Jackson Immunoresearch, used at 1∶200. Wing discs were mounted in VECTASHIELD Mounting Media (Vector Laboratories, Inc. #H-1000). Fluorescence images were obtained on a Leica SP5 confocal microscope or on a LSM 510 Zeiss confocal microscope. The NIH Image J program was used to perform measurements. Quantifications of the intensity of Caspase 3 signals were performed as described in [21]. Quantifications of the ratio of Phalloidin signal between posterior and anterior wing compartments were performed as described in [7]. To quantify the ratio of Cpa or Cpb signals between the anterior and posterior wing disc compartments, a region of interest (ROI) of 100 per 50 pixels was selected. The sum of the gray values was measured for each ROI, applied to each compartments for each disc on optical cross sections through distal wing disc epithelium comprising the apical surface. To measure wing size, wing were dissected one to two days after eclosion and imaged using the Hamamatsu Orca-ER camera attached to a Zeiss' Stereo Lumar V12 stereoscope. The total area of each wing was outlined and measured using the *area measurement* function. Statistical significance was calculated using a two-tailed *t*-test.

### Western Blotting

For each genetic background, proteins were extracted from either four wing imaginal discs or four dechorionated embryos using a 2x SDS sample buffer (Sigma #S3401). Samples were frozen in liquid nitrogen, boiled for 5 minutes in 5 µl Sample Buffer 2x, spun at 13,000 g for 1 minute, loaded on a 10% SDS-PAGE gel and transferred to a PVDF membrane (Amersham Hybond-P, GE Healthcare). Proteins were visualized by immunoblotting using rabbit anti-Cpa (1∶2500) or rabbit anti-Cpb (1∶2500) or mouse anti-HA (Covance 11 MMS101P; 1∶1000) or rabbit anti-Histone H3 (Cell Signalling #9715; 1∶3000). HRP-conjugated donkey anti-mouse or donkey anti-rabbit secondary antibodies were used at 1∶5000 (Jackson ImmunoResearch Laboratories, Inc.). Blots were developed using Amersha ECL Plus Western Blotting Detection System (GE Healthcare). Densitometric analysis of signal intensity was performed using the GelQuant.NET software (biochemlabsolutions.com) and normalized with the loading control. Statistical significance was calculated using a *Paired t-test*.

### Isolation of RNA and Real-Time qRT-PCR

Total RNAs were extracted from either 10 first instar larvae or 50 wing imaginal discs for each genetic background. Samples were homogenized in RLT buffer treated with DNase (Qiagen) at 4 degree C and total RNAs were isolated using the RNeasy mini kit (Qiagen) following manufacturer instructions. First Strand cDNA Synthesis Kit for RT-PCR (Roche) was used to produce cDNAs from 1 µg of total RNA. To quantify mRNA levels, qPCRs were carried out on reverse-transcribed total mRNA using intron-exon-specific primers (Table S1), designed using the Primer3 software [38], [39], and ensuring that efficiency is at least 90% and restricting primer dimmer formation. Real-time qPCR was performed using PerfeCTa SYBR Green FastMix (Quanta Biosciences) in 384 well skirted PCR microplates (Axygen) sealed with optically clear sealing tape (STARSTEDT) in the Applied Biosystems 7900HT Fast Real-Time PCR System. The relative amount of mRNA for each condition was calculated after normalization to the *RpL32* transcript. Statistical significance was calculated using a *Paired t-test* with significance at *P*<*0.05*.

## Results

### Cpa and Cpb stabilize each other's protein levels and accumulate at Adherens Junctions

**Figure 1:**
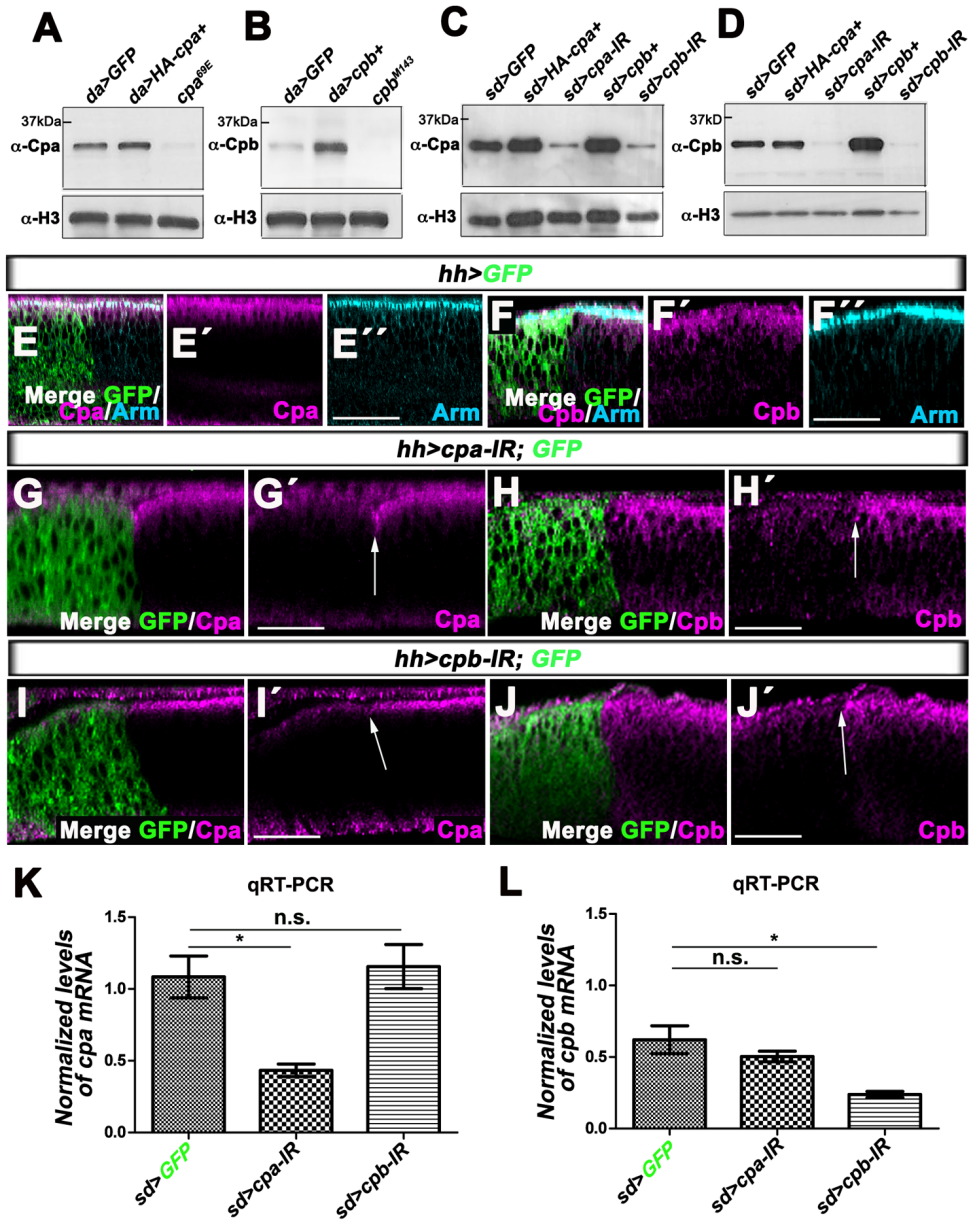
Loss of *cpa* or *cpb* reduces both Cpa and Cpb protein levels. (A) western blot on protein extracts from embryos expressing UAS-*mCD8GFP* (lane 1) or *UAS-HA-cpa^89E^* (lane 2) under *da*-Gal4 control or homozygote mutant for the *cpa^69E^* allele (lane 3), blotted with anti-Cpa (upper panel) and anti-H3 (lower panel). (B) western blot on protein extracts from embryos expressing UAS-*mCD8GFP* (lane 1) or UAS-*cpb^7^* (lane 2) under *da*-Gal4 control or homozygote mutant for the *cpb^M143^* allele (lane 3), blotted with anti-Cpb (upper panel) and anti-H3 (lower panel). (C and D) western blots on protein extracts from wing imaginal discs expressing UAS-*mCD8GFP* (lane 1) or UAS-*HA-cpa^89E^* (lane 2) or UAS-*cpa-IR^C10^* (lane 3) or UAS-*cpb^7^* (lane 4) or UAS-*cpb-IR^45668^* (lane 5) under *sd*-Gal4 control, blotted with (C) anti-Cpa (upper panel) and anti-H3 (lower panel) or (D) anti-Cpb (upper panel) and anti-H3 (lower panel). (E–E″ to J–J′) optical cross sections through distal wing disc epithelium of third instar larvae with apical side up in which *hh*-Gal4 drives (E–E″ and F–F″) UAS-*mCD8-GFP* (green in E and F) and (G–G′ and H–H′) UAS-*cpa-IR^C10^* or (I–I′ and J–J′) UAS-*cpb-IR^45668^*. Discs are stained with (E–E″, G–G′ and I–I′) anti-Cpa (magenta) or (F–F″, H–H′ and J–J′) anti-Cpb (magenta) and (E–E″ and F–F″) anti-Arm. The arrows in G′, H′, I′ and J′ mark the limits of the posterior compartment boundary. The scale bars represent 15 µm. (K and L) graphs of (K) *cpa* or (L) *cpb* mRNA levels measured by five independent qRT-PCR in wing imaginal discs expressing UAS-*mCD8GFP* (lane 1) or UAS-*cpa-IR^C10^* (lane 2) or UAS-*cpb-IR^45668^* (lane 3) under *sd*-Gal4 control. (K) the mean for *sd>GFP* is 1.084; for *sd>cpa-IR^C10^* is 0.4328; for *sd>cpb-IR^45668^* is 1.155. *P<0.0027* for comparison of lane 1 and 2. (L) the mean for *sd>GFP* is 0.6210; for *sd>cpa-IR^C10^* is 0.5037; for *sd>cpb-IR^45668^* is 0.2375. *P<0.0049* for comparison of lane 1 and 3. n.s. indicates a non-significant *P*. Error bars indicate s.e.m.

**Figure 2:**
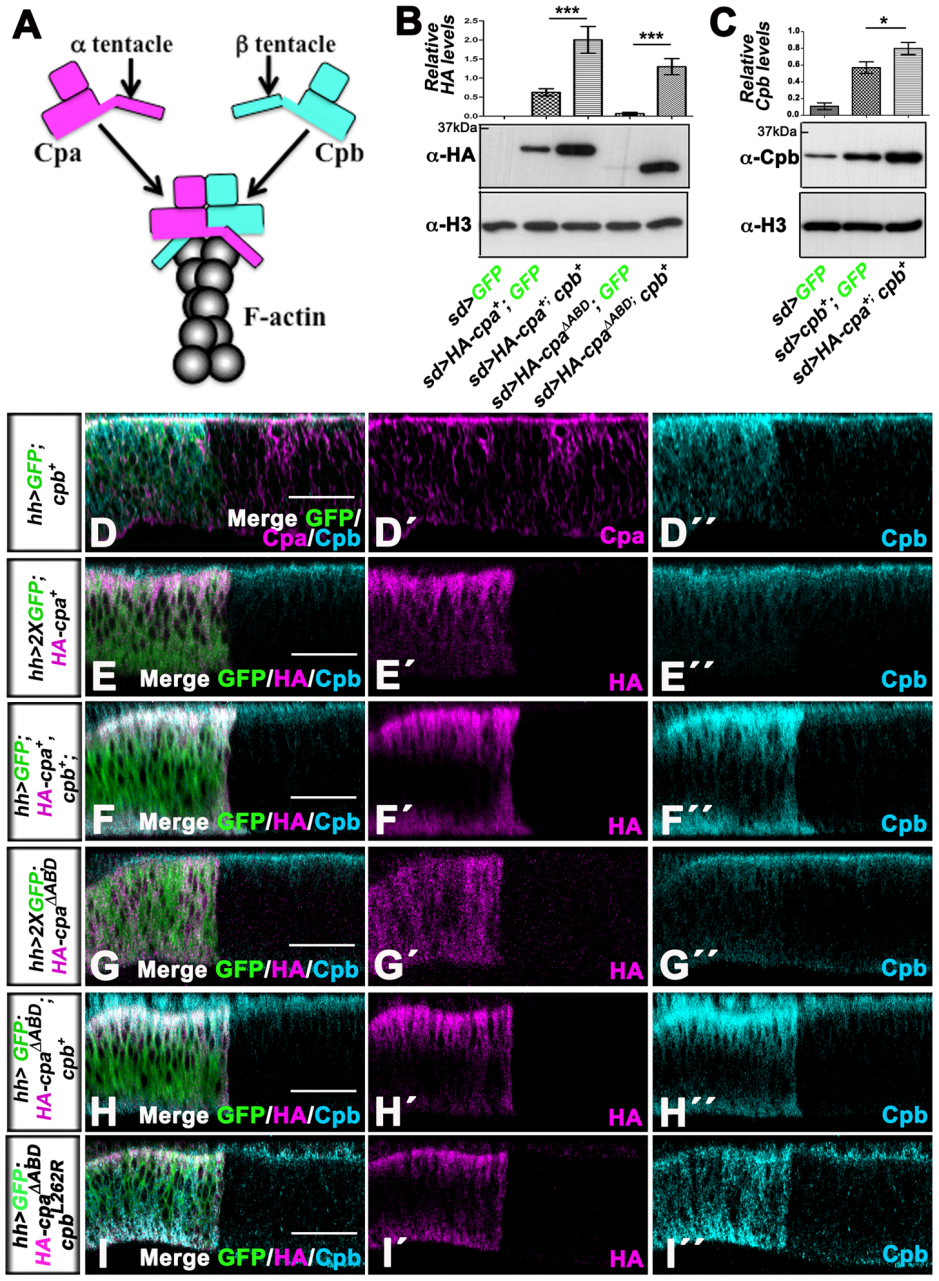
Increasing the levels of individual CP subunits alone has no effect on the endogenous levels of the other subunit, while co-expressing *HA-cpa* or *HA-cpa^ΔABD^* and *cpb* enhance synergistically the levels of both subunits. (A) model by which Cpa and Cpb cap F-actin barbed ends via the interaction of α and β tentacles with actin. (B) western blot on protein extracts from wing discs expressing UAS-*mCD8-GFP* (lane 1) or UAS-*mCD8-GFP* and UAS-*HA-cpa^89E^* (lane 2) or UAS-*HA-cpa^89E^* and UAS*-cpb^7^* (lane 3) or UAS-*mCD8-GFP* and UAS-*HA-cpa^ΔABD^* (lane 4) or UAS-*HA-cpa^ΔABD^* and UAS-*cpb^7^* (lane 5) under *sd*-Gal4 control, blotted with anti-HA (middle panel) and anti-H3 (lower panel). The means for lane 1 is 0, for lane 2 is 0.6250, for lane 3 is 2, for lane 4 is 0.0667, for lane 5 is 1.300. Error bars indicate s.e.m.. *P<0.0092* for comparison of lanes 2 and 3 and of lanes 4 and 5. (B) western blot on protein extracts from wing discs expressing UAS-*mCD8-GFP* (lane 1) or UAS-*mCD8-GFP* and UAS-*cpb^7^* (lane 2) or UAS-*cpb^7^* and UAS-*HA-cpa^89E^* (lane 3) under *sd*-Gal4 control, blotted with anti-Cpb (middle panel) and anti-H3 (lower panel). The upper panels in B and C represent a quantification of relative (B) HA or (C) Cpb intensity signals for each genetic combination, measured by 4 independent blots. The means for lane 1 is 0.1088, for lane 2 is 0.5699, for lane 3 is 0.7982. Error bars indicate s.e.m. *P<0.0182* for comparison of lanes 2 and 3. (D–D″ to I–I″) optical cross sections through distal epithelia of third instar wing imaginal discs with apical sides up and posterior sides to the left in which *hh*-Gal4 drives (D–D″) UAS-*cpb^7^* and one copy of UAS-*mCD8-GFP* (green in D) or (E–E″) UAS-*HA-cpa^89E^* and two copies of UAS-*mCD8-GFP* (green in E) or (F–F″) UAS-*HA-cpa^89E^*, UAS*-cpb^7^* and one copy of UAS-*mCD8-GFP* (green in F) or (G–G″) UAS-*HA-cpa^ΔABD^* and two copies of UAS-*mCD8-GFP* (green in G) or (H–H″) UAS-*HA-cpa^ΔABD^*, UAS*-cpb^7^* and one copy of UAS-*mCD8-GFP* (green in H) or (I–I″) UAS-*cpb^L262R^*, UAS-*HA-cpa^ΔABD^* and one copy of UAS-*mCD8-GFP* (green in I). Discs are stained with anti-Cpb (Cyan blue) and (D–D″) anti-Cpa (magenta) or (E–E″ to I–I″) anti-HA (magenta), which reveals (E–E″ and F–F″) *HA-cpa^89E^* or (G–G″ to I–I″) *HA-cpa^ΔABD^* expression. The scale bars represent 15 µm.

To understand how Cpa and Cpb are regulated to restrict growth of *Drosophila* epithelia, we generated polyclonal antibodies to each CP subunit. In lysates from embryos expressing UAS-*mCD8-GFP* under the control of the ubiquitous *daughterless*-Gal4 (*da*-Gal4) driver, the Cpa (Fig. 1A) and Cpb (Fig. 1B) antibodies revealed a band at around 32 and 31 kDa respectively by Western Blot. These signals were lost in embryonic extracts from homozygous *cpa* (Fig. 1A) or *cpb* mutants (Fig. 1B) respectively. Conversely, overexpressing full length *cpa*, tagged with HA (UAS-*HA-cpa^+^*; Fig. 1A) or *cpb* (UAS*-cpb^+^*; Fig. 1B) with *da*-Gal4, enhanced the anti-Cpa or anti-Cpb signals respectively. Similarly, Cpa levels were increased in wing disc lysates overexpressing *HA-cpa* under *scalloped*-Gal4 control (*sd>HA-cpa^+^*; Fig. 1C), while endogenous Cpb levels were similar to control *sd>GFP* lysates (Fig. 1D). Forcing *cpb* expression in this tissue also induced a significant increase in Cpb levels by Western Blot (Fig. 1D) but did not significantly affect endogenous Cpa levels (Fig. 1C). Cross-sections through wing disc epithelia expressing UAS*-mCD8-GFP* in the posterior compartment using the *hedgehog*-Gal4 (*hh*-Gal4) driver showed that Cpa (Fig. 1E–E′′′) and Cpb (Fig. 1F–F′′′) accumulated at the apical cell membrane and co-localized with components of Adherens Junctions, including the β-Catenin homolog Armadillo (Arm). Co-expressing *cpb* and *mCD8-GFP* in this domain strongly enhanced the anti-Cpb signals but did not affect Cpa levels (Fig. 2D–D″). Conversely, *hh>HA-cpa^+^* wing disc epithelia displayed an apical localization of HA-Cpa, like endogenous Cpa (Fig. 1E–E″), but no change in Cpb levels (Fig. 2E–E″). Thus, the anti-Cpa and Cpb antibodies recognize specifically Cpa and Cpb respectively.

Strikingly, Cpa levels were strongly reduced not only in wing disc extracts expressing double-stranded RNAs (dsRNA) for *cpa* under *sd*-Gal4 control (*sd>cpa-IR)* but also in discs knocked-down for *cpb* (*sd>cpb-IR*; Fig. 1C). In the converse experiment, the amount of Cpb was also strongly reduced in both *sd>cpb-IR* and *sd>cpa-IR* wing disc extracts (Fig. 1D). Similarly, knocking down *cpa* (Fig. 1G–G′ and H–H′) or *cpb* (Fig. 1I–I′ and J–J′) in the posterior wing disc compartment with *hh*-Gal4 significantly reduced the apical accumulation of both Cpa and Cpb when compared to anterior compartments used as internal controls. Moreover, both Cpa and Cpb levels were also strongly reduced in lysates from first instar larvae homozygote mutant for *cpa* or *cpb* (Fig. S1A) and in clones mutant for *cpa* or *cpb* (Fig. S1B–B″ to E–E″). To verify that the *cpa* dsRNA did not affect *cpb* mRNA and *vice versa*, we performed quantitative RT-PCR (qRT-PCR) experiments on wing imaginal discs knocked down for *cpa* or *cpb*. As expected, *sd>cpa-IR* or *sd>cpb-IR* wing discs showed a significant reduction of *cpa* (Fig. 1K, 2.5±0.43 folds) or *cpb* mRNA (Fig. 1L, 2.6±0.41 folds) levels respectively, relative to control *sd>GFP*. However, *cpa* mRNA levels were not significantly affected by a reduction in *cpb* (Fig. 1K), nor were *cpb* mRNA levels reduced in wing discs knocked-down for *cpa* (Fig. 1L). Similarly, a reduction in *cpa* or *cpb* levels had no effect on *cpb* or *cpa* mRNA levels, respectively, in first instar larvae expressing *cpa-IR* or *cpb-IR* under *da*-Gal4 control (Fig. S1F and G). Taken together, we conclude that Cpa and Cpb accumulate at apical cell membrane and enhance each other's protein levels.

### Cpa and Cpb levels are rate limited to form a functional heterodimer

**Figure 3:**
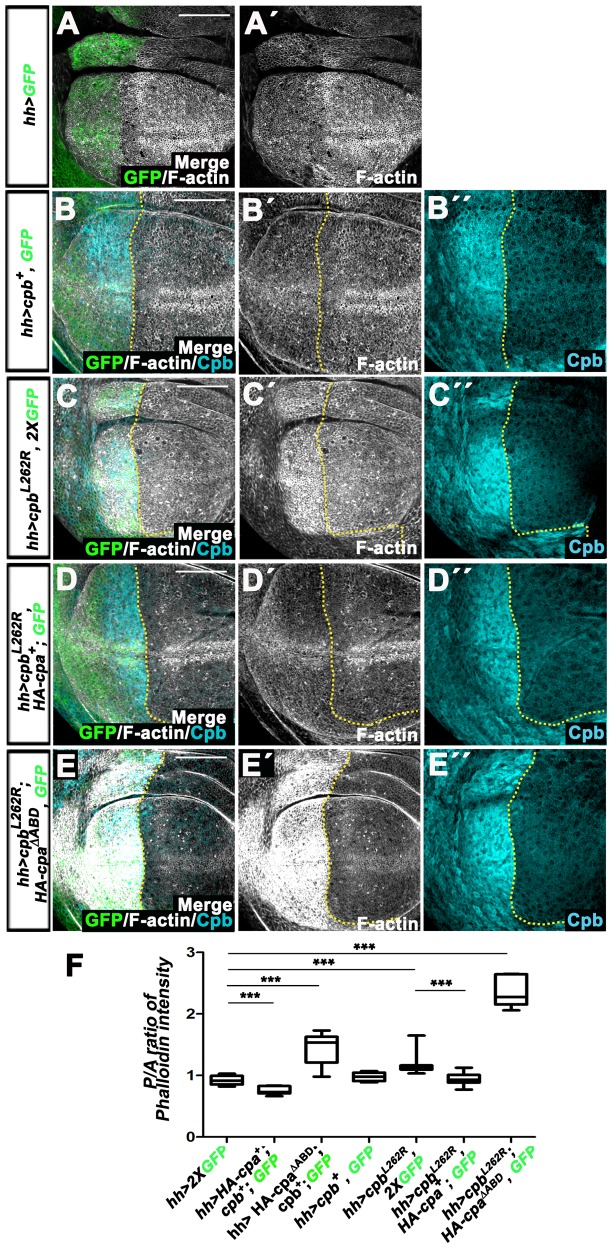
Overexpressing *HA-cpa* suppresses the apical F-actin accumulation of *cpb^L262R^*-expressing wing discs, whereas *HA-cpa^ΔABD^* expression has the opposite effect. (A–A″ to E–E″) standard confocal sections of the apical cell membrane of third instar wing imaginal discs with dorsal sides up and posterior sides to the left, expressing (A–A′) one copy of UAS-*mCD8-GFP* (green in A) or (B–B″) UAS-*cpb^7^* and one copy of UAS-*mCD8-GFP* (green in B) or (C–C″) UAS-*cpb^L262R^* and two copies of UAS-*mCD8-GFP* (green in C) or (D–D″) UAS-*cpb^L262R^*, UAS-*HA-cpa^89E^* and one copy of UAS-*mCD8-GFP* (green in D) or (E–E″) UAS-*cpb^L262R^*, *UAS-HA-cpa^ΔABD^* and one copy of UAS-*mCD8-GFP* (green in E) under *hh*-Gal4 control. Discs are stained with Phalloidin (white) to mark F-actin and (B–B″ to E–E″) anti-Cpb (cyan blue). The yellow lines outline the anterior-posterior compartment boundary. The scale bars represent 30 µm. (F) Mean intensity of the ratio of Phalloidin signal between posterior and anterior wing compartments of *hh*-Gal4 driving two copies of UAS-*mCD8-GFP* (lane 1) or UAS-*HA-cpa^89E^*, UAS-*cpb^7^* and one copy of UAS-*mCD8-GFP* (lane 2) or UAS-*HA-cpa^ΔABD^*, UAS*-cpb^7^* and one copy of UAS-*mCD8-GFP* (lane 3) or UAS-*cpb^7^* and one copy of UAS-*mCD8-GFP* (lane 4) or UAS-*cpb^L262R^* and two copies of UAS-*mCD8-GFP* (lane 5) or UAS-*cpb^L262R^*, UAS-*HA-cpa^89E^* and one copy of UAS-*mCD8-GFP* (lane 6) or UAS-*cpb^L262R^*, UAS-*HA-cpa^ΔABD^* and one copy of UAS-*mCD8-GFP* (lane 7). The mean for lane 1 is 0.922 (n = 12) for lane 2 is 0.775 (n = 8), for lane 3 is 1.435 (n = 10), for lane 4 is 0.977 (n = 10), for lane 5 is 1.175 (n = 16), for lane 6 is.0.937 (n = 14), for 7 is 2.348 (n = 6). Error bars indicate s.e.m.. *** indicate *P<0.0001*.

The Capping Protein α and β subunits form a functional heterodimer, which caps F-actin barbed ends via the interaction of the α and β tentacles with actin (Fig. 1A and [11], [12], [13], [20]). To confirm that the stabilization of Cpa and Cpb's protein levels by each other promotes the formation of a functional heterodimer, we first tested if co-expressing *cpb* and *HA-cpa* would enhance the levels of both subunits by comparing the levels of HA-Cpa and Cpb when overexpressed alone or together, ensuring that each genetic combination contained the same number of UAS transgenes. Indeed, by Western Blot (Fig. 2B, *P* *<0.0092*) and in wing disc epithelia (Fig. 2 compare F–F″ with E–E″), HA levels were strongly enhanced when *HA-cpa* was co-expressed with *cpb*. Similarly, the co-expression of *HA-cpa* and *cpb* strongly increased Cpb levels compared to wing disc lysates overexpressing *cpb* alone (Fig. 2C). Overexpressed *HA-cpa* and *cpb* appeared to form a functional heterodimer as their co-expression in the posterior wing disc compartment with *hh*-Gal4 decreased the apical F-actin ratio between both compartments compared to *hh>GFP* control (Fig. 3F, *P* *<0.0001*). In contrast, overexpressing either *HA-cpa* or *cpb* alone has no effect on F-actin levels [21]. We conclude that the levels of endogenous Cpa and Cpb available are rate limited to form a functional heterodimer.

### Forms of CP mutated in α or β tentacle counteract the ability of wild type CP to restrict F-actin accumulation

Surprisingly, expressing an HA-tagged form of Cpa deleted of the α tentacle (UAS-*HA-cpa^ΔABD^*) has no significant effect on F-actin when expressed alone [21] but triggered apical F-actin accumulation when co-expressed with *cpb* (Fig. 3F, *P* *<0.0001* and [21]), indicating that HA-Cpa^ΔABD^ affects F-actin only in the presence of overexpressed *cpb*. We therefore tested if the co-expression of *cpb* would also enhance the levels of HA-Cpa^ΔABD^. In contrast to full length HA-Cpa, which accumulated apically (Fig. 2E–E″), HA-Cpa^ΔABD^ localized uniformly along the apical-basal axis in the posterior compartment of *hh>HA-cpa^ΔABD^* wing discs (Fig. 2G–G″). Strikingly, co-expressing *cpb* not only enhanced strongly HA-Cpa^ΔABD^ levels as assessed by Western Blot (Fig. 2B, *P* *<0.0002*), but also relocalized HA-Cpa^ΔABD^ at the apical cell membrane (Fig. 2H–H″). Thus, forcing Cpb levels enhances the levels of HA-Cpa^ΔABD^ and promotes its apical localization.

The heterodimer formed between HA-Cpa^ΔABD^ and Cpb appears to have reduced capping activity and may be recruited to F-actin barbed ends, preventing the binding of wild type CP. If so, we would expect that a form of Cpb truncated of its β tentacle would also promote F-actin accumulation in the presence of endogenous CP. To test this possibility, we expressed a form of *cpb* mutated in the highly conserved Leucine 262 (UAS-*cpb^L262R^*), which has been proposed to directly interact with actin [12]. While overexpressing full length *cpb* had no significant effect on F-actin (Fig. 3 compare B–B″ with A–A′ and F), *hh>cpb^L262R^* wing discs accumulated apical F-actin in the posterior compartment (Fig. 3C–C″ and F, *P<0.0001*). However, co-expressing full length *HA-cpa* in these tissues suppressed the apical F-actin accumulation due to the presence Cpb^L262R^ (Fig. 3D–D″ and F, *P<0.0001*). Thus, forcing Cpa levels tethers the effects of Cpb^L262R^ on F-actin. In contrast, F-actin accumulation was strongly enhanced when *cpb^L262R^* was co-expressed with *HA-cpa^ΔABD^* (Fig. 3E–E″ and F, *P<0.0001*). Moreover, Cpb^L262R^, like full length Cpb, enhances HA-Cpa^ΔABD^ levels and triggered its relocalization to the apical cell membrane (Fig. 2I–I″). We conclude that forms of CP with reduced capping activity inhibit wild type CP to restrict F-actin accumulation, most likely by tethering barbed ends, preventing the recruitment of wild type CP.

### CP and forms of CP with dominant negative effects on F-actin have opposite effects on tissue growth

**Figure 4:**
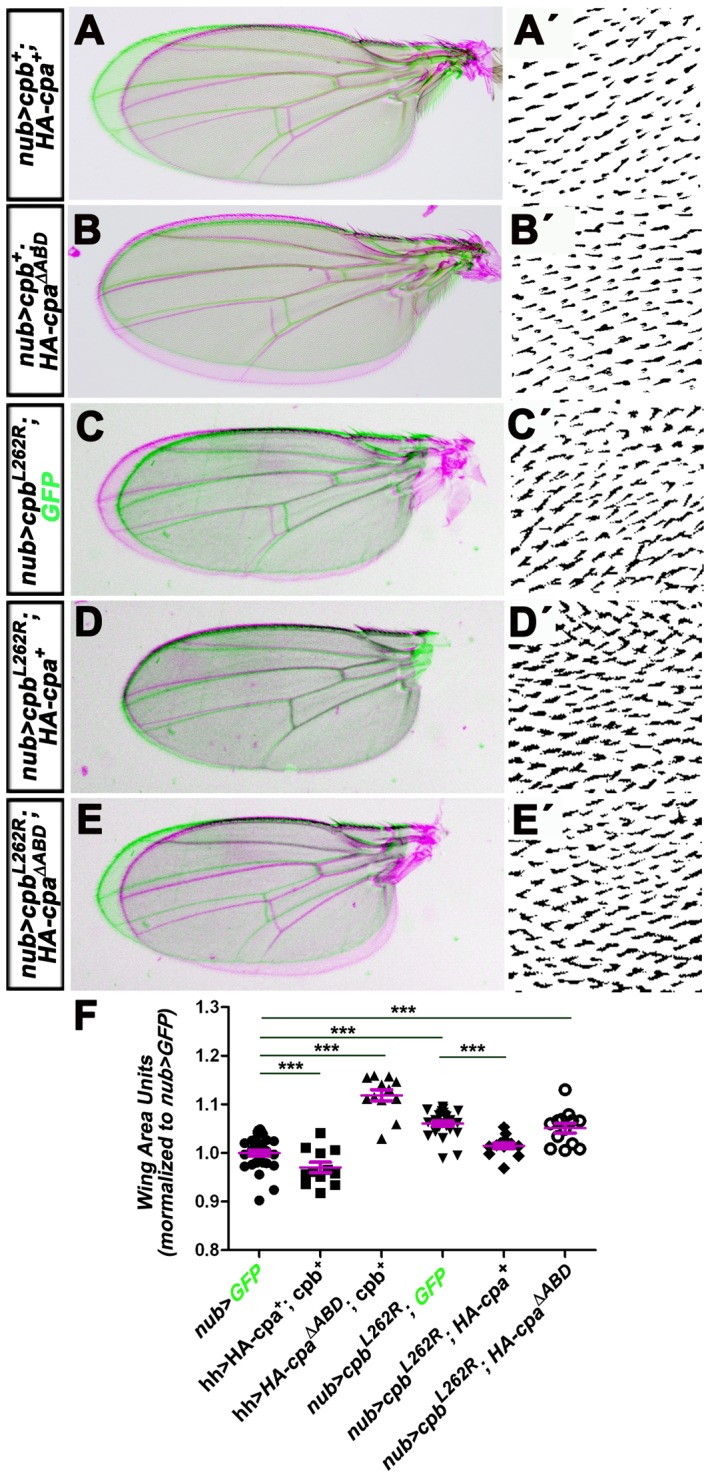
Overexpressing full length *HA-cpa* and *cpb* prevents wing growth, while ectopic expression of *HA-cpa^ΔABD^* and/or *cpb^L262R^* has the opposite effect. (A, B, C, D and E) merge between adult wings expressing in green UAS*-mCD8GFP* under *nub*-Gal4 control and in magenta (A) UAS-*HA-cpa^89E^* and UAS-*cpb^7^* or (B) UAS-*HA-cpa^ΔABD^* and UAS-*cpb^7^* or (C) UAS-*cpb^L262R^* and one copy of UAS-*mCD8-GFP* or (D) UAS-*cpb^L262R^* and UAS*-HA-cpa^89E^* or (E) UAS-*cpb^L262R^* and UAS-*HA-cpa^ΔABD^* under *nub*-Gal4 control. (A′, B′, C′ D′ and E′) magnification of hairs on adult wings for the genotypes shown in A, B, C, D and E. (F) quantification of relative wing size normalized to *nub>GFP* control for *nub*-Gal4 driving UAS-*mCD8-GFP* (lane 1) or UAS-*HA-cpa^89E^* and UAS-*cpb^7^* (lane 2) or UAS-*HA-cpa^ΔABD^* and UAS-*cpb^7^* (lane 3) or UAS-*cpb^L262R^* and one copy of UAS-*mCD8-GFP* (lane 4) or UAS-*cpb^L262R^* and UAS-*HA-cpa^89E^* (lane 5) or UAS-*cpb^L262R^* and UAS-*HA-cpa^ΔABD^* (lane 6). The mean for lane 1 is 1(n = 32), for lane 2 is 0.9702 (n = 12), for lane 3 is 1.119 (n = 13), for lane 4 is 1.061 (n = 24), for lane 5 is 1.015 (n = 13), for lane 6 is 1.051 (n = 13). Error bars indicate s.e.m.. *P<0.015* for comparison of lanes 1 and 2. *P<0.0001* for comparison of lanes 1 and 3 or 4 or 6 and for comparison of lane 4 and 5.

Decreasing or increasing CP levels has opposite effects on F-actin levels (Fig. 3F and [25]). Because loss of *CP* induces overgrowth of the wing disc epithelium by promoting Yki activity [7], [9], we asked of overexpressing *cpa* and *cpb* has an opposite effect on tissue growth. Indeed, overexpressing full length *HA-cpa* and *cpb* in the wing primordium using the *nubbin*-Gal4 (*nub*-Gal4) driver significantly reduced the size of the adult wing (Fig. 4A, compare *nub>GFP* control wing in green to *nub>cpa^+^, cpb^+^* wing in magenta and F; *P<0.0151*), but does not affect cell survival [21]. Thus, tight CP levels are critical to control tissue growth.

To determine if CP controls tissue growth via F-actin regulation, we analyzed the effect of expressing forms of *cpa* and *cpb* that have dominant negative effects on F-actin on wing growth. Expressing *HA-cpa^ΔABD^* and *cpb* (Fig. 4B and F, *P* *<0.0001*) or *cpb^L262R^* alone (Fig. 4C and F, *P* *<0.0001*) or combined with *HA-cpa^ΔABD^* (Fig. 4E and F, *P* *<0.0001*) under *nub*-Gal4 control, not only promoted apical F-actin accumulation (Fig. 3), but also enhanced significantly the growth of adult wings. Strikingly, expressing *HA-cpa* suppressed the overgrowth of *nub>cpb^L262R^* wings (Fig. 4D and F, *P* *<0.0001*), indicating that the effect of Cpb^L262R^ on F-actin and tissue growth is dependent on the levels of full length Cpa. Because altering the levels or activity of CP did not affect the density of wing hairs (Fig. 4A′, B′, C′ D′ and E′), which develop from one single cell, the CP-dependent growth defects most likely result from changes in proliferation rate rather than alteration of cell size. We conclude that a CP-dependent reduction of F-actin levels correlates with tissue undergrowth, while a CP-dependent increase in F-actin levels is associated with tissue overgrowth.

### The α tentacle is not absolutely required to form a functional heterodimer

**Figure 5:**
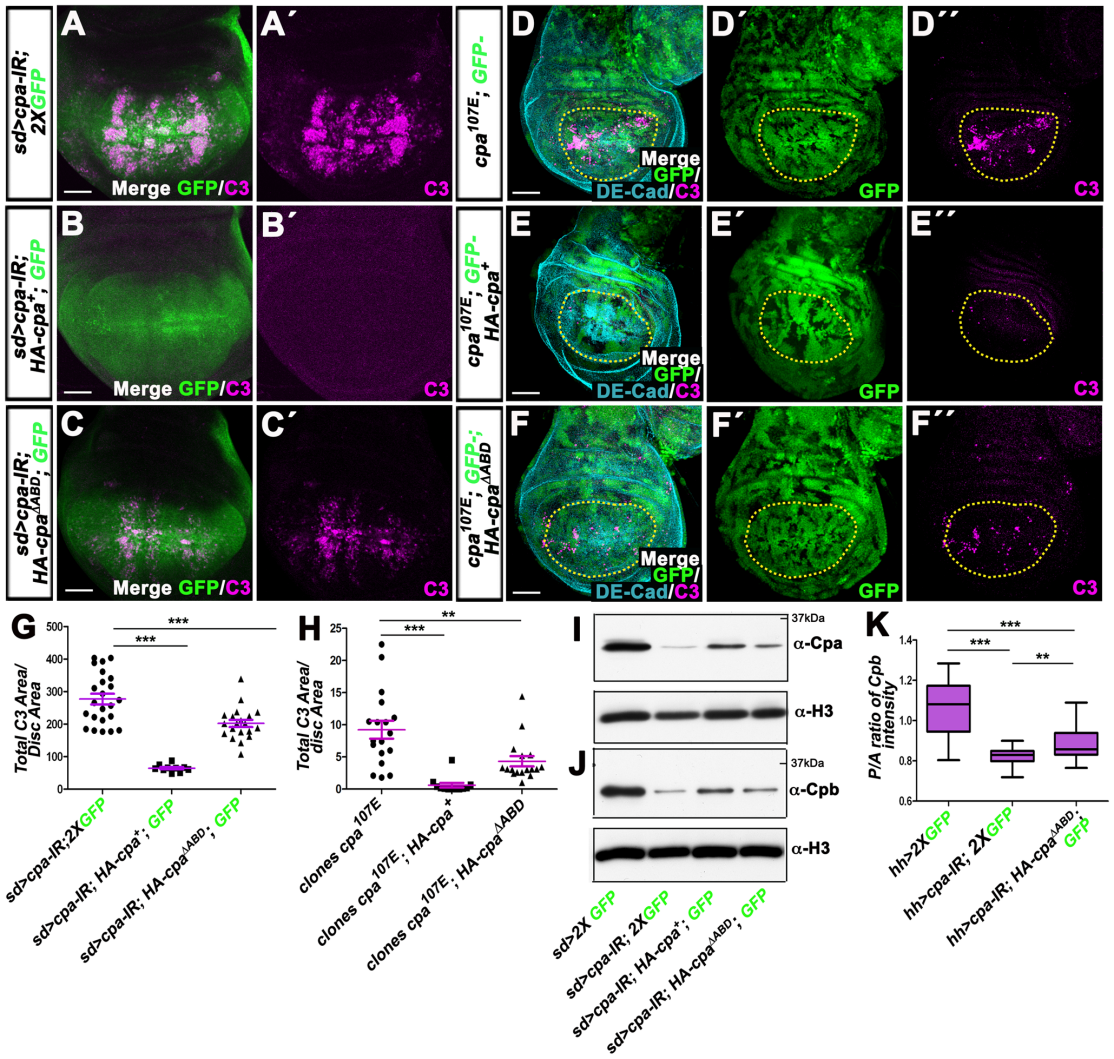
Expressing *HA-cpa* or *HA-cpa^ΔABD^* suppresses apoptosis and restores Cpb levels of wing discs knocked-down for *cpa*. (A–A′ to F–F′) standard confocal sections of third instar wing imaginal discs with dorsal sides up. (A–A′ to C–C′) *sd*-Gal4 driving (A–A′) UAS-*cpa-IR^C10^* and two copies of UAS-*mCD8-GFP* (green in A) or (B–B′) UAS-*cpa-IR^C10^*, UAS-*HA-cpa^89E^* and one copy of UAS-*mCD8-GFP* (green in B) or (C–C′) UAS-*cpa-IR^C10^*, UAS-*HA-cpa^ΔABD^* and one copy of UAS-*mCD8-GFP* (green in C). (D–D″ to F–F″) *T155*-Gal4; UAS-*flp* induced *cpa^107E^* mutant clones marked by the absence of GFP (green) and expressing (E–E″) UAS-*HA-cpa^89E^* or (F–F″) UAS-*HA-cpa^ΔABD^* in the whole wing disc epithelium. Discs are stained with anti-activated-Caspase 3 (magenta), which monitors DRONC activation and (D–D″ to F–F″) anti-DE-Cad (cyan blue). The scale bars represent 30 µm. (G) quantification of total C3 area per disc area for the three genotypes shown in A–A′ to C–C′. The mean for *sd>cpa-IR^C10^, 2XGFP* is 92.4 (n = 23); for *sd>cpa-IR^C10^, HA-cpa^89E^*, *1XGFP* is 0.7 (n = 10); for *sd>cpa-IR^C10^, HA-cpa^ΔABD^*, *1XGFP* is 51.4 (n = 20). Error bars indicate s.e.m. *P<0.0001* for comparison of lane 1 and 2. *P<0.0005* for comparison of lane 1 and 3. (H) quantification of total C3 area per disc area for the three genotypes shown in D–D″ to F–F″. The means for *T155>flp; cpa^107E^* is 9.228 (n = 18); for *T155>flp; cpa^107E^*; UAS-*HA-cpa^89E^* is 0.608 (n = 12); for *T155>flp; cpa^107E^*; UAS-*HA-cpa^ΔABD^* is 4.329 (n = 17). Error bars indicate s.e.m. *P<0.0001* for comparison of *T155>flp; cpa^107E^* and *T155>flp; cpa^107E^*; UAS-*HA-cpa^89E^* and *P<0.0048* for comparison of *T155>flp; cpa^107E^* and *T155>flp; cpa^107E^*; UAS-*HA-cpa^ΔABD^*. (I and J) western blots on protein extracts from wing discs expressing two copies of UAS-*mCD8-GFP* (lane 1) or UAS*-cpa-IR^C10^* and two copies of UAS-*mCD8-GFP* (lane 2) or UAS-*cpa-IR^C10^* and UAS-*HA-cpa^89E^* and one copy of UAS-*mCD8-GFP* (lane 3) or UAS*-cpa-IR^C10^* and UAS-*HA-cpa^ΔABD^* and one copy of UAS-*mCD8-GFP* (lane 4) under *sd*-Gal4 control, blotted with (I) anti-Cpa (upper panel) and anti-H3 (lower panel) or (J) anti-Cpb (upper panel) and anti-H3 (lower panel). (K) mean intensity of the ratio of Cpb intensity signals between posterior and anterior wing compartments of *hh*-Gal4 driving two copies of UAS-*mCD8-GFP* (lane 1) or UAS-*cpa-IR^C10^* and two copies of UAS-*mCD8-GFP* (lane 2) or UAS-*cpa-IR^C10^* and UAS-*HA-cpa^ΔABD^* and one copy of UAS-*mCD8-GFP* (lane 3). The mean for lane 1 is 1.064 (n = 20), for lane 2 is 0.822 (n = 17), for lane 3 is 0.883 (n = 24). Error bars indicate s.e.m.. *P<0.0001* for comparison of lanes 1 and 2 or 3 or *P<0.0085* for comparison of lanes 2 and 3.

Because the heterodimer formed between HA-Cpa^ΔABD^ and Cpb appears to be recruited at F-actin barbed ends, we tested if HA-Cpa^ΔABD^ can partially compensate for the loss of endogenous Cpa. Expressing *cpa-IR* under *sd*-Gal4 control induced the activation of Caspase 3 in numerous cells in the distal wing disc epithelium (Fig. 5A–A′). Apoptosis was almost fully suppressed by overexpressing full length *HA-cpa* (Fig. 5B–B′ and G; *P<0.0001*). Expressing *HA-cpa^ΔABD^* also significantly prevented apoptosis of *sd>cpa-IR* wing discs, although to a much weaker extent than *HA-cpa* (Fig. 5C–C′ and G; *P<0.0005*). These effects were not only due to titration of the *cpa* dsRNAs by the overexpressed *cpa* constructs as *HA-cpa* (Fig.5E–E″ and H) or *HA-cpa^ΔABD^* (Fig. 5F–F″ and H; *P<0.0048*) also rescued apoptosis of clones mutant for a *cpa* allele. Expressing *HA-cpa* or *HA-cpa^ΔABD^* in *sd>cpa-IR* wing discs also partially restored Cpa (Fig. 5I) and Cpb (Fig. 5J) levels, as assessed by Western blot. Quantification of the ratio of Cpb signals between the posterior and anterior compartments of wing discs expressing *cpa-IR* under *hh*-Gal4 control showed that knocking-down *cpa* reduced Cpb levels in the posterior compartment compared to *hh>GFP* control (Fig. 5K). This decrease in Cpb levels was significantly alleviated by the presence of HA-Cpa^ΔABD^ (Fig. 5K *P<0.0085*). We conclude that in the absence of wild type Cpa, Cpa^ΔABD^ s capable of forming a functional heterodimer with Cpb, which prevents apoptosis.

### Cpb compensates for a reduction in *cpa* by enhancing *cpa* mRNA levels and *vice versa*

**Figure 6:**
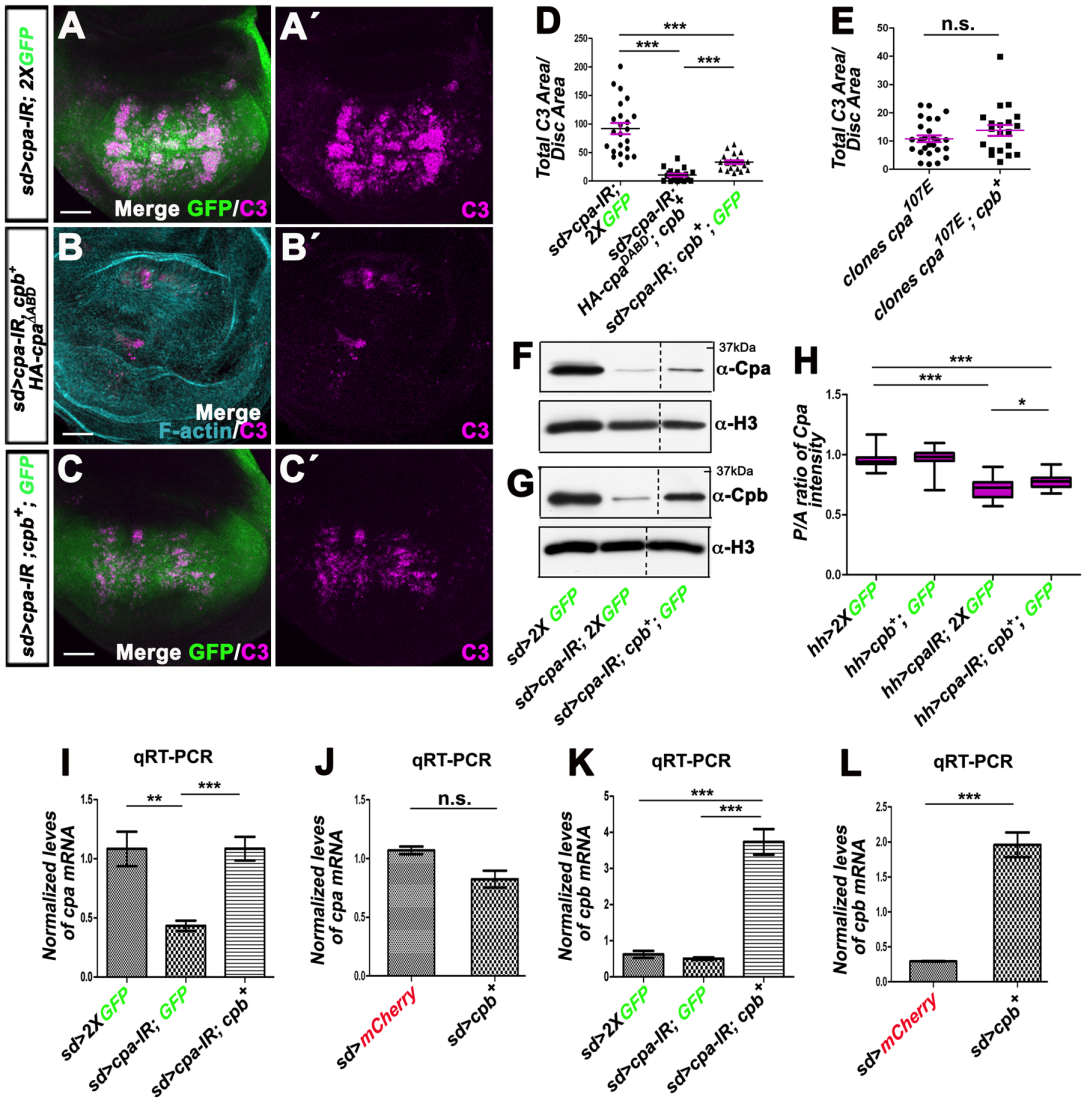
Overexpressing *cpb* in wing discs knocked-down for *cpa*, restores *cpa* mRNA and protein levels and suppresses apoptosis. (A–A′ to C–C′) standard confocal sections of third instar wing imaginal discs with dorsal sides up, expressing (A–A″) UAS-*cpa-IR^C10^* and two copies of UAS-*mCD8-GFP* (green in A) or (B–B′) UAS-*cpa-IR^C10^*, UAS-*HA-cpa^ΔABD^* and UAS-*cpb^7^* or (C–C′) UAS-*cpa-IR^C10^*, UAS-*cpb^7^* and one copy of UAS-*mCD8-GFP* (green in C) under *sd*-Gal4 control. Discs are stained with anti-activated-Caspase 3 (magenta), which monitors DRONC activation and (B–B′) Phalloidin (cyan blue in B) to underline wing disc shape. The scale bars represent 30 µm. (D) quantification of total C3 area per disc area for the genotypes *sd>cpa-IR^C10^, 2XGFP* (lane 1); *sd>cpa-IR^C10^*, *HA-cpa^ΔABD^*, *cpb^7^* (lane 2) and *sd>cpa-IR^C10^*, *cpb^7^*, *1XGFP* (lane 3). The means for lane 1 is 92.4 (n = 23); for lane 2 is 10.61 (n = 19); for lane 3 is 32.9 (n = 20). Error bars indicate s.e.m. *P<0.0001* for comparison of lane 1 and 2 or 3 or lane 2 and 3. (E) quantification of total C3 area per disc area for wing discs containing *T155>flp; cpa^107E^* mutant clones (lane 1) or *T155>flp; cpa^107E^* mutant clones expressing UAS-*cpb^7^* (lane 2). The means for lane 1 is 10.80 (n = 26); for lane 2 is 13.77 (n = 20). n.s. indicates non-significant *P* value. (F and G) western blots on protein extracts from wing discs expressing two copies of UAS-*mCD8-GFP* (lane 1) or UAS*-cpa-IR^C10^* and two copies of UAS-*mCD8-GFP* (lane 2) or UAS*-cpa-IR^C10^*, UAS-*cpb^7^* and one copy of UAS-*mCD8-GFP* (lane 3) under *sd*-Gal4 control, blotted with (F) anti-Cpa (upper panel) and anti-H3 (lower panel) or (G) anti-Cpb (upper panel) and anti-H3 (lower panel). Panels derive from the same experiment shown in Figure 5E and F and blots were processed in parallel (see Figure S2 showing the whole experiment). (H) mean intensity of the ratio of Cpa signals between posterior and anterior wing compartments of *hh*-Gal4 driving two copies of UAS-*mCD8-GFP* (lane 1) or UAS-*cpb^7^* and one copy of UAS-*mCD8-GFP* (lane 2) or UAS-*cpa-IR^C10^* and two copies of UAS-*mCD8-GFP* (lane 3) or UAS-*cpa-IR^C10^* and UAS-*cpb^7^* and one copy of UAS-*mCD8-GFP* (lane 4). The mean for lane 1 is 0.959 (n = 15), for lane 2 is 0.970 (n = 20), for lane 3 is 0.716 (n = 21), for lane 4 is 0.776 (n = 20). Error bars indicate s.e.m.. *P<0.0001* for comparison of lanes 1 and 3 or 4 or *P<0.01* for comparison of lanes 3 and 4. (I to L) graph of (I and J) *cpa* or (K and L) *cpb* mRNA levels measured by five independent qRT-PCR in wing imaginal discs expressing (I and K) two copies of UAS-*mCD8GFP* (lane 1) or UAS-*cpa-IR^C10^* and UAS-*mCD8GFP* (lane 2) or UAS-*cpa-IR^C10^* and UAS*-cpb^7^* (lane 3) or (J and L) UAS-*mCherry* (lane 1) or UAS-*cpb^7^* (lane 2) under *sd*-Gal4 control. (I) the means for lane 1 is 1.084; for lane 2 is 0.4328; for lane 3 is 1.086. *P<0.0027* for comparison of lane 1 with 2 or *P<0.0003* for comparison of lane 2 with 3. (J) the means for lane 1 is 1.07; for lane 2 is 0.824. n.s. indicates non-significant *P* value. (K) the means for lane 1 is 0.621; for lane 2 is 0.5031; for lane 3 is 3.735. *P<0.0001* for comparison of lane 3 with 1 or 2. (L) the means for lane 1 is 0.292; for lane 2 is 1.961. *P<0.0001* for comparison of lane 1 and 2. Error bars indicate s.e.m.

**Figure 7:**
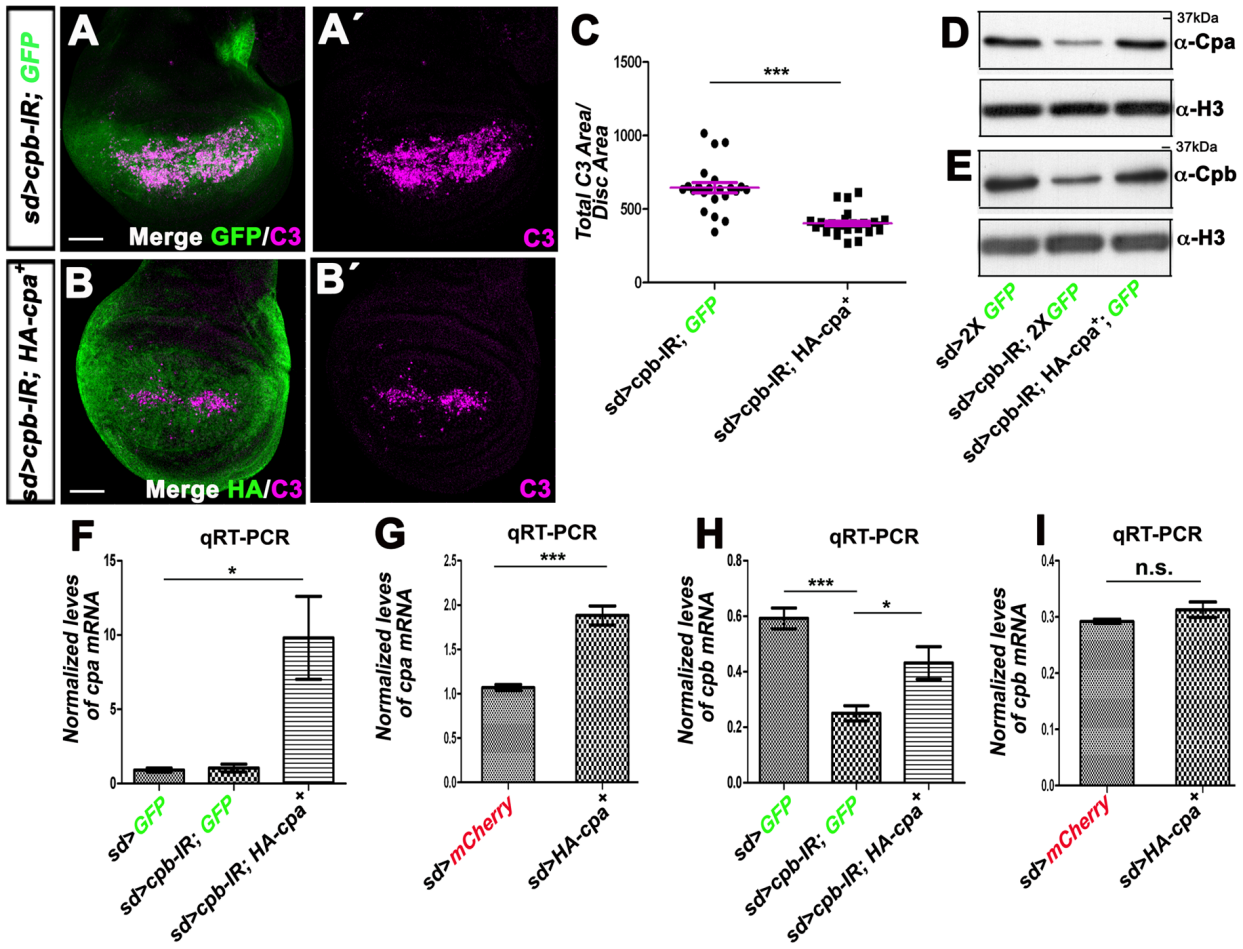
Overexpressing *HA-cpa* in wing discs knocked-down for *cpb* restores *cpb* mRNA and protein levels and suppresses apoptosis. (A–A′ and B–B′) standard confocal sections of third instar wing imaginal discs with dorsal sides up, expressing (A–A″) UAS-*cpb-IR^45668^* and one copy of UAS-*mCD8-GFP* (green in A) or (B–B′) UAS-*cpb-IR^45668^* and UAS-*HA-cpa^89E^* under *sd*-Gal4 control. Discs are stained with anti-activated-Caspase 3 (magenta), which monitors DRONC activation and (B–B′) anti-HA (green in B), reflecting *HA-cpa^89E^* expression. The scale bars represent 30 µm. (C) quantification of total C3 area per disc area for the two genotypes shown in A–A′ and B–B′. The means for *sd>cpb-IR^45668^, GFP* is 62.19 (n = 22); for *sd>cpb-IR^4566^*, *HA-cpa^89E^* is 26.67 (n = 32). Error bars indicate s.e.m. *P<0.0001* for comparison of between both genotypes. (D and E) western blots on protein extracts from wing discs expressing two copies of UAS-*mCD8-GFP* (lane 1) or UAS*-cpb-IR^45668^* and two copies of UAS-*mCD8-GFP* (lane 2) or UAS-*cpb-IR^45668^* and UAS-HA-*cpa^89E^* and one copy of UAS-*mCD8-GFP* (lane 3) under *sd*-Gal4 control, blotted with (D) anti-Cpa (upper panel) and anti-H3 (lower panel) or (E) anti-Cpb (upper panel) and anti-H3 (lower panel). (F to I) graphs of (F and G) *cpa* or (H and I) *cpb* mRNA levels measured by (F and H) three or (G and I) five independent qRT-PCR in wing imaginal discs expressing (F and H) two copies of UAS-*mCD8GFP* (lane 1) or UAS-*cpb-IR^45668^* and UAS-*mCD8GFP* (lane 2) or UAS-*cpb-IR^45668^* and UAS-*HA-cpa^89E^* (lane 3) or (G and I) UAS-*mCherry* (lane 1) or UAS-*HA-cpa^89E^* under *sd*-Gal4 control. (F) the means for lane 1 is 0.90; for lane 2 is 1.04; for lane 3 is 9.8. *P<0.033* for comparison of lane 1 with 3. (G) the means for lane is 1.07; for lane 2 is 1.88. *P<0.0001* for comparison of lane 1 and 2. (H) the means for lane 1 is 0.59; for lane 2 is 0.25; for lane 3 is 0.4319. *P<0.0018* for comparison of lane 1 and 2 or *P<0.048* for comparison of lane 2 and 3. (I) the means for lane 1 is 0.29; for lane 2 is 0.31. n.s. indicates non-significant *P* value. Error bars indicate s.e.m.

Interestingly, co-expressing *cpb* with *HA-cpa^ΔABD^* almost fully suppressed apoptosis of wing discs knocked-down for *cpa* (Fig. 6 compare B–B′ with A–A′ and D; *P<0.0001*). This effect could be due to the stabilization and apical relocalization of HA-Cpa^ΔABD^ when co-expressed with *cpb* (Fig. 2H–H″). However, apoptosis of *sd>cpa-IR* wing discs was also significantly suppressed by overexpressing *cpb* alone (Fig. 6C–C′ and D; *P<0.0001*). Conversely, expressing *HA-cpa* in tissues knocked-down for *cpb* (*sd>cpb-IR*) also prevented apoptosis (Fig. 7 compare B–B′ with A–A′ and C; *P<0.0001*).

To understand the mechanisms by which Cpa and Cpb compensate for each other's function, we tested the effect of overexpressing *cpb* on Cpa levels in *cpa*-depleted tissues. As previously observed, by Western Blots, Cpa (Fig. 6F) and Cpb (Fig. 6G) levels were strongly reduced in wing disc extracts knocked-down for *cpa*. Forcing *cpb* levels in these tissues enhanced the levels of both Cpa (Fig. 6F and Fig. S2) and Cpb (Fig. 6G and Fig. S2). We quantified this effect by measuring the ratio of Cpa signals between the posterior and anterior compartments of *hh>cpa-IR*-expressing wing discs, in the presence or absence of overexpressed *cpb*. While in control *hh>GFP* tissues this ratio was 0.95, knocking down *cpa* reduced this ratio to 1,34 folds (Fig. 6H; *P<0.0001*). This effect was significantly alleviated by the overexpression of *cpb* (Fig. 6H; *P<0.01*). In contrast, overexpressing *cpb* in control *hh>GFP* wing discs did not affect Cpa levels (Fig. 6H), indicating that Cpb enhances Cpa levels only when cells contain reduced Cpa levels. By Western Blots, *HA-cpa* also enhanced both Cpa (Fig. 7D) and Cpb (Fig. 7E) levels when expressed in tissues knocked-down for *cpb*. Thus, Cpa compensates for a reduction in *cpb* by stimulating the production of Cpb, and *vice versa*.

Using qRT-PCR, we next analyzed if overexpressing either subunits affects the mRNA levels of the other. After normalization to the *RpL32* transcript used as an internal control, we observed that whereas *cpa* (Fig. 6I, *P* *<0.0027*) but not *cpb* (Fig. 6K) mRNA levels were strongly reduced in wing discs knocked-down for *cpa* (*sd>cpa-IR*), forcing *cpb* levels in these tissues fully restored *cpa* mRNA to wild type levels (Fig. 6I; *P<0.0003*). In contrast, in wing discs that contained endogenous *cpa* and *cpb*, overexpressing *cpb*, which strongly enhanced *cpb* mRNA levels (Fig. 6L), had no significant effect on *cpa* mRNA levels (Fig. 6J). Thus, Cpb stimulates the production or stabilization of *cpa* mRNA only when Cpa levels are reduced. In the converse experiment, overexpressing *HA-cpa* in *sd>cpb*-depleted wing discs enhanced the levels of both *cpa* (Fig. 7F) and *cpb* (Fig. 7H; *P<0.0018*) mRNA. However, in wing discs that contained endogenous *cpa* and *cpb*, only *cpa* mRNA levels were strongly increased (Fig. 7G and I). The ability of Cpb to suppress apoptosis of *cpa*-depleted wing discs was due to the increase in *cpa* mRNA and protein levels as clones mutant for a *cpa* allele showed similar apoptotic levels in the absence or presence of overexpressing *cpb* (Fig. 6E). We conclude that Cpa compensates for a reduction in *cpb* by increasing *cpb* mRNA levels and *vice versa*.

## Discussion

### Cpa and Cpb regulate each other at multiple levels

Our data argue that in *Drosophila*, different pools of Cpa and/or Cpb co-exist, and they regulate each other at various levels. One level of regulation involves their reciprocal stabilization of their protein levels. First, in *Drosophila*, like in yeast, the loss of one CP subunit reduces the protein levels of the other subunit ([26] and Fig. 1) but does not affect its mRNA levels (Fig. 1 and Fig. S1). Second, co-expressing *cpa* and *cpb* in *Drosophila* tissues enhances synergistically the levels of both subunits relative to the levels of each subunit overexpressed alone (Fig. 2). Third, large quantities of soluble active chicken CP can be produced in bacteria only when both subunits are co-expressed [40]. Cpa and Cpb may stabilize each other's protein levels via direct protein-protein interactions [19]. The tight interaction between both subunits may prevent the recruitment of E3 ubiquitin ligases that would otherwise target individual CP subunits for degradation by the 26S proteasome. As an heterodimer, CP has been shown to bind to the fast polymerizing ends of actin filaments, preventing further addition of actin monomers [41], [42] and to restrict F-actin accumulation in *Drosophila* tissues [25], [27]. In addition, Cpa and Cpb appear to show some function on their own as overexpressing *cpb* rescues apoptosis of wing discs knocked-down for *cpa* and *vice versa* (Fig. 6 and 7). Overexpression of *cpb* alone is also sufficient to enhance the retinal defects of flies knocked down for the Cbl-interacting protein *cindr* [43] and to rescue the migration and F-actin polarization defects of *Drosophila* border cells mutant for *warts* [44]. Because individual chicken CP subunits expressed in bacteria are mainly deposited into insoluble cytoplasmic inclusion bodies but can be renaturated as active heterodimers [45], individual subunit may exist in the cell as pools of insoluble monomers. The molecular mechanism by which individual CP subunit compensates for each other's function remains to be determined. Several observations argue that this mechanism involves the production of the subunit knocked-down by the other subunit via an increase of its mRNA levels (Fig. 6 and 7). CP has been observed in the nuclei of chicken retinal and kidney epithelial cells in culture, in Madin-Darby canine kidney (MDCK) cells, in *Xenopus laevis* oocytes and bovine lens epithelial cells in culture [46], [47]. Whether Cpa and Cpb influence each other's transcription in the nucleus is an interesting possibility to be tested. The protein-mRNA feedbacks between Cpa and Cpb may guarantee that a pool of functional heterodimer is present to limit F-actin polymerization. However, a CP-dependent negative feedback mechanism must exist that restricts the production of CP in excess, as forcing the expression of one of the subunit in tissues that contain endogenous CP does not enhance the mRNA and protein levels of the other subunit (Fig. 6 and 7). Because the loss of one subunit has no effect on the mRNA levels of the other subunit (Fig. 1 and Fig. S1), the CP-dependent negative feedback may act by limiting the ability of individual subunits to stimulate the production of each other's mRNAs. Thus, in addition to regulate each other's protein levels, individual CP subunit stimulates each other's mRNA production up to an optimal physiological threshold of functional heterodimers. Further experiments are necessary to elucidate the protein-mRNA feedback loop mechanisms, which operate between both subunits.

### Capping activity of the CP heterodimer at actin filament barbed ends

Our observations argue that *in vivo* the actin-binding domain of Cpa is not absolutely required to form a functional CP heterodimer, as HA-Cpa^ΔABD^ partially compensates for the loss of endogenous Cpa (Fig. 5). Consistent with our observations, in actin assembly assays, a mutant form of the chicken α subunit that lacks the α tentacle is able to cap F-actin [12]. Nevertheless, the α tentacle may favor the interaction and therefore stabilization of the α subunit by the β. This possibility is consistent with the observation that HA-Cpa^ΔABD^ is found in the cell at much lower levels than full length HA-Cpa (Fig. 2) despite both transgenes being inserted at the same locus in the fly genome and therefore likely expressed at similar levels [21]. Consistent with this hypothesis, Arginine 259 of the chicken α1 tentacle forms side-chain hydrogen bonds with three residues of the β subunit, all residues being conserved across isoforms and species [19]. Moreover, *in vitro*, a truncated form of the chicken α1 subunit, consisting only of the C-terminal domain, retains the ability to form a heterodimer [48]. The reduced ability of HA-Cpa^ΔABD^ to interact with Cpb may explain its inability to fully suppress apoptosis of Cpa-depleted tissues (Fig. 5) and to affect F-actin levels when overexpressed alone [21]. However, several observations indicate that the α and β tentacles also enable full capping activity *in vivo*. First, in actin assembly assays, the C-terminus of the chicken α1 and β1 subunits are required for high-affinity capping [12]. Second, in the presence of endogenous CP, stabilizing HA-Cpa^ΔABD^ levels by forcing *cpb* expression does not reduce F-actin levels, as does overexpressed *HA-cpa/cpb*, but instead, promotes F-actin accumulation (Fig. 3 and [21]). Third, replacing leucine 262 of the chicken β subunit has no effect on protein stability and global structure but decreases the capping affinity significantly [12], [20]. Fourth, identical mutations in the β orthologs induces F-actin accumulation in *Drosophila* tissues (Fig. 3) and disrupts the sarcomere of mouse heart [24]. Thus, we propose that the heterodimers formed between HA-Cpa^ΔABD^ and Cpb or between Cpb^L262R^ and Cpa are recruited to F-actin barbed ends and cap actin filaments less efficiently than wild type CP. The low capping activity of the HA-Cpa^ΔABD^/Cpb heterodimer is sufficient to partially compensate for the loss of Cpa. However, in the presence of endogenous CP, the HA-Cpa^ΔABD^/Cpb heterodimers compete with wild type Cpa/Cpb heterodimers for binding the barbed ends of F-actin, which can lead to defects in F-actin.

### Tight regulation of CP levels is critical to control tissue growth

CP appears to act as a gatekeeper, which limits the development of cancer-related processes. Loss of the α subunit promotes Yki/YAP/TAZ-dependent proliferation in *Drosophila* epithelia and in human cells [9], [31], causes a significantly increase in gastric cancer cell migration and is associated with cancer-related death [10]. In contrast, increasing CP levels has opposite effects: it reduces tissue growth (Fig. 4) and prevents Src-mediated tumour development in *Drosophila* [21], and significantly restricts gastric cancer cell migration [10]. Several of our observations argue that the function of CP on tissue growth involves its F-actin capping activity. First expressing *cpb^L262R^*, which contains a single point mutation affecting the capping activity [23], induces F-actin accumulation (Fig. 3) and wing overgrowth (Fig. 4). Moreover, CP-dependent F-actin accumulation correlates with tissue overgrowth, whereas tissue undergrowth is associated with a CP-dependent reduction in F-actin (Fig. 3 and 4). Consistent with these observations, other actin regulators have been shown to control Yki/YAP/TAZ dependent tissue growth [7], [9], [31]. Thus, a reduction or an increase of CP levels has deleterious consequences on tissue growth, implying that it must be tightly regulated. This may be achieved in part by the ability of Cpa and Cpb to stimulate or limit the production of each other in conditions of lower or higher CP levels respectively, assuring that a pool of functional CP heterodimer is produced in sufficient quantities in the cell to prevent cancer development but not in excess to sustain proper tissue growth.

## Supporting Information

Figure S1 **Reducing** ***cpa*** **or** ***cpb*** **levels reduces both Cpa and Cpb protein levels.** (**A**) western blot on protein extracts from first instar larvae, either *white minus* (lane 1) or homozygote mutant for *cpa^69E^* (lane 2) or homozygote mutant for *cpb^M143^* (lane 3), blotted with (upper panel) anti-Cpa (upper bands) and anti-Cpb (lower band) and (lower panel) anti-H3. (B–B″ to E–E″) standard confocal sections of third instar wing imaginal discs, containing (B–B″ and C–C″) *T155*-Gal4; UAS-*flp* induced *cpa^69E^* mutant clones marked by the absence of GFP (green) or (D–D″ and E–E″) heat shocked-induced *cpb^M143^* mutant clones marked by the absence of GFP (green). Discs are stained with (B–B″ and E–E″) anti-Cpa (magenta) or (C–C″ and E–E″) anti-Cpb (magenta). The scale bars represent 15 µm. (F and G) graphs of (F) *cpa* or (G) *cpb* mRNA levels measured by three independent qRT-PCR in first instar larvae expressing UAS-*mCD8-GFP* (lane 1) or UAS-*cpa-IR^C10^* (lane 2) or UAS-*cpb*-IR^45668^ (lane 3) under *da*-Gal4 control. (F) The means for lane 1 is 7.04; for lane 2 is 1.13; for lane 3 is 5.91. Error bars indicate s.e.m.. *P<0.015* for comparison of lane 1 and 2. (F) The means for lane 1 is 1.97; for lane 2 is 1.96; for lane 3 is 0.46. Error bars indicate s.e.m.. *P<0.021* for comparison of lane 1 and 3. n.s. indicates non-significant *P* values.
(TIF)

Figure S2 **Expressing** ***HA-cpa*** **or** ***HA-cpa^ΔABD^*** **or** ***cpb*** **in wing discs knocked down for** ***cpa*** **restores Cpa and Cpb levels.** Western blots on protein extracts from wing discs expressing two copies of UAS-*mCD8-GFP* (lane 1) or UAS*-cpa-IR^C10^* and two copies of UAS-*mCD8-GFP* (lane 2) or UAS*-cpa-IR^C10^* and UAS-*HA-cpa^89E^* and one copy of UAS-*mCD8-GFP* (lane 3) or UAS*-cpa-IR^C10^* and UAS-*HA-cpa^ΔABD^* and one copy of UAS-*mCD8-GFP* (lane 4) or UAS*-cpa-IR^C10^* and UAS-*HA-cpa^ABD^*, which contains the last 28 amino acids of the Cpa C-terminus and one copy of UAS-*mCD8-GFP* (lane 5) or UAS-*cpa-IR^C10^*, UAS-*cpb^7^* and one copy of UAS-*mCD8-GFP* (lane 6) under *sd*-Gal4 control, blotted with (A) anti-Cpa (upper panel) and anti-H3 (lower panel) or (B) anti-Cpb (upper panel) and anti-H3 (lower panel).
(TIF)

Table S1 **Intron-exon-specific primers used to quantify** ***cpa*** **,** ***cpb*** **and** ***RpL32*** **mRNA levels by qRT-PCR.**
(DOCX)
