# Correction: Subunits of the *Drosophila* Actin-Capping Protein Heterodimer Regulate Each Other at Multiple Levels

**DOI:** 10.1371/journal.pone.0102614

**Published:** 2014-07-07

**Authors:** The PLOS ONE Staff

## Notice of Republication

This article was republished on June 20, 2014, due to a typesetting error. The publisher apologizes for this error. Please download the PDF again to view the corrected article. The originally published, uncorrected article and the republished, corrected article are provided here for reference.

## Supporting Information

File S1 Originally published, uncorrected article
(PDF)

File S2 Republished, corrected article
(PDF)
