# Supplementary figures and images for: Subunits of the Drosophila Actin-Capping Protein Heterodimer Regulate Each Other at Multiple Levels
Source: PLoS One. 2014 May 2;9(5):e96326. doi: 10.1371/journal.pone.0096326 (PMC4008575; doi:10.1371/journal.pone.0096326)

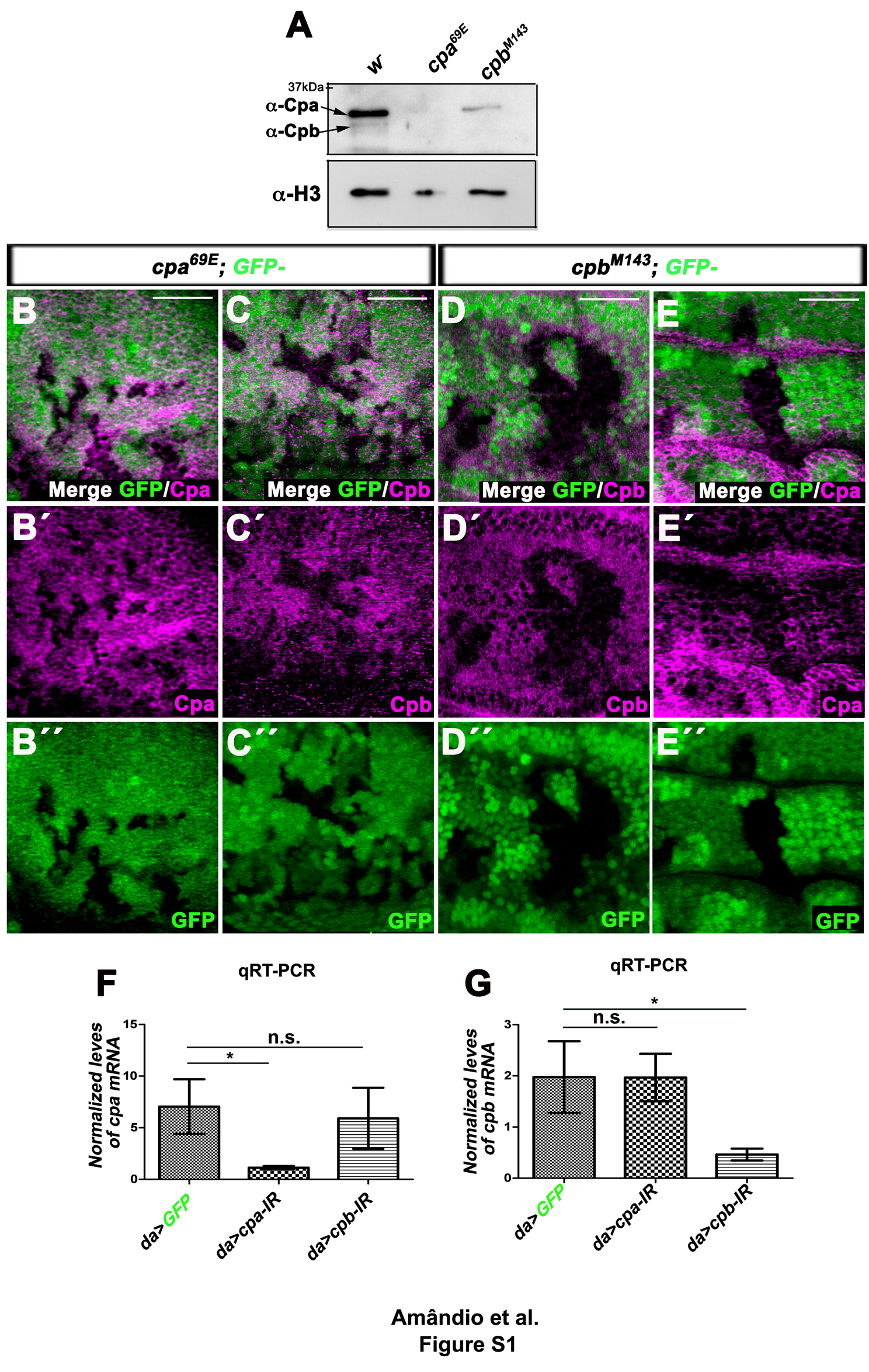

Supplement: Figure S1 — Reducing cpa or cpb levels reduces both Cpa and Cpb protein levels. (A) western blot on protein extracts from first instar larvae, either white minus (lane 1) or homozygote mutant for cpa69E (lane 2) or homozygote mutant for cpbM143 (lane 3), blotted with (upper panel) anti-Cpa (upper bands) and anti-Cpb (lower band) and (lower panel) anti-H3. (B–B″ to E–E″) standard confocal sections of third instar wing imaginal discs, containing (B–B″ and C–C″) T155-Gal4; UAS-flp induced cpa69E mutant clones marked by the absence of GFP (green) or (D–D″ and E–E″) heat shocked-induced cpbM143 mutant clones marked by the absence of GFP (green). Discs are stained with (B–B″ and E–E″) anti-Cpa (magenta) or (C–C″ and E–E″) anti-Cpb (magenta). The scale bars represent 15 µm. (F and G) graphs of (F) cpa or (G) cpb mRNA levels measured by three independent qRT-PCR in first instar larvae expressing UAS-mCD8-GFP (lane 1) or UAS-cpa-IRC10 (lane 2) or UAS-cpb-IR45668 (lane 3) under da-Gal4 control. (F) The means for lane 1 is 7.04; for lane 2 is 1.13; for lane 3 is 5.91. Error bars indicate s.e.m.. P<0.015 for comparison of lane 1 and 2. (F) The means for lane 1 is 1.97; for lane 2 is 1.96; for lane 3 is 0.46. Error bars indicate s.e.m.. P<0.021 for comparison of lane 1 and 3. n.s. indicates non-significant P values. (TIF) [file pone.0096326.s001.tif]

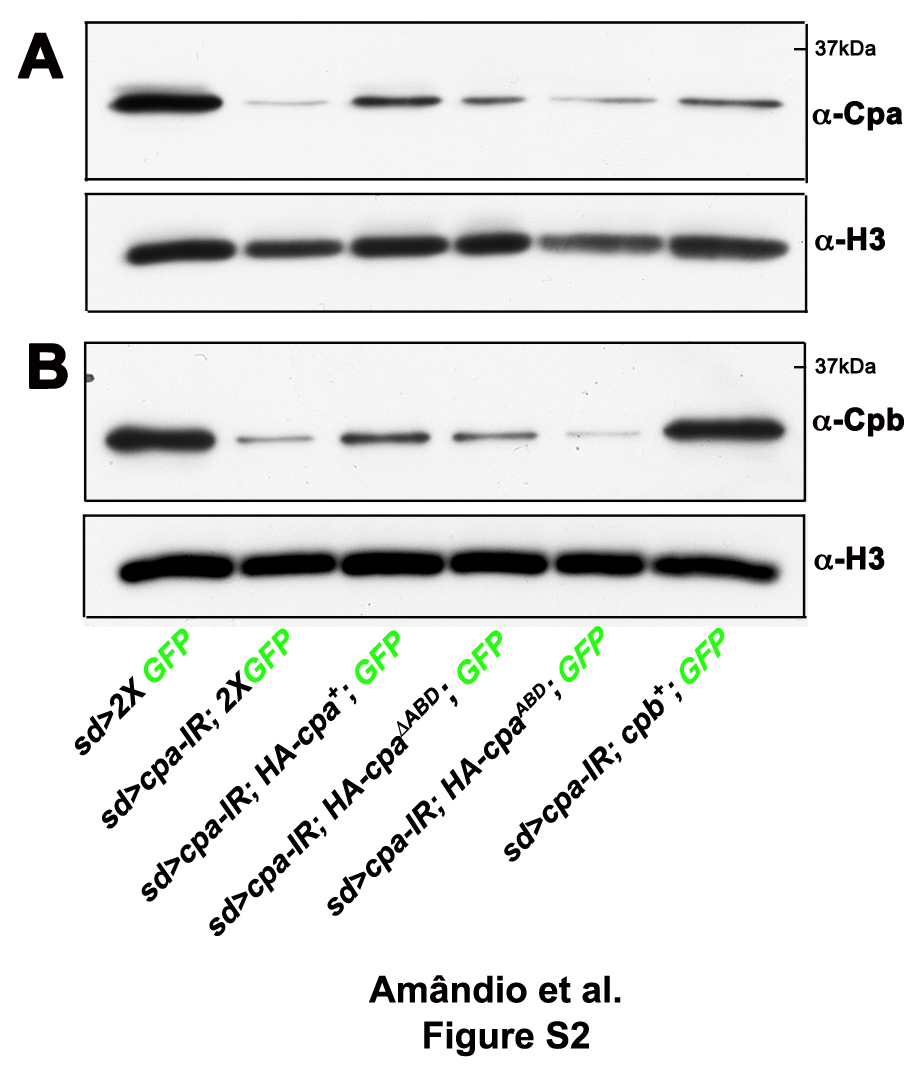

Supplement: Figure S2 — Expressing HA-cpa or HA-cpaΔABD or cpb in wing discs knocked down for cpa restores Cpa and Cpb levels. Western blots on protein extracts from wing discs expressing two copies of UAS-mCD8-GFP (lane 1) or UAS-cpa-IRC10 and two copies of UAS-mCD8-GFP (lane 2) or UAS-cpa-IRC10 and UAS-HA-cpa89E and one copy of UAS-mCD8-GFP (lane 3) or UAS-cpa-IRC10 and UAS-HA-cpaΔABD and one copy of UAS-mCD8-GFP (lane 4) or UAS-cpa-IRC10 and UAS-HA-cpaABD, which contains the last 28 amino acids of the Cpa C-terminus and one copy of UAS-mCD8-GFP (lane 5) or UAS-cpa-IRC10, UAS-cpb7 and one copy of UAS-mCD8-GFP (lane 6) under sd-Gal4 control, blotted with (A) anti-Cpa (upper panel) and anti-H3 (lower panel) or (B) anti-Cpb (upper panel) and anti-H3 (lower panel). (TIF) [file pone.0096326.s002.tif]
