# Supplementary material for: Subunits of the Drosophila Actin-Capping Protein Heterodimer Regulate Each Other at Multiple Levels
Source: PLoS One. 2014 May 2;9(5):e96326. doi: 10.1371/journal.pone.0096326 (PMC4008575; doi:10.1371/journal.pone.0096326)
Supplement: Table S1 — Intron-exon-specific primers used to quantify cpa , cpb and RpL32 mRNA levels by qRT-PCR. (DOCX) [file pone.0096326.s003.docx]

**Supplementary Table 1: Primers used to produce cDNAs for *cpa*, *cpb* and the control RpL32.**

| Cpa Fw | GAGAAGGTGCGCATCGTATC |
| --- | --- |
| Cpa Rev | AGCCCCATCCTTTAGCAGAG |
| Cpb Fw | TGTGCTTTGGATCTGATGCG |
| Cpb Rev | TCCTTGTCCTTGGCGATCTT |
| Rpl32 FW | CCGCTTCAAGGGACAGTATC |
| Rpl32 Rev | CAATCTCCTTGCGCTTCTTG |
| Mp20 exon1 Fw | TGGCATCAGTGTTAGCTTGTCA |
| Mp20 intron1 Fw- | TTTCTTATCCGATCCACCTAG |
| Mp20exon2 Rev- | GTTGGCAATATCCTTCTTCTCG |
