# Supplementary material for: Correction: Subunits of the Drosophila Actin-Capping Protein Heterodimer Regulate Each Other at Multiple Levels
Source: PLoS One. 2014 Jul 7;9(7):e102614. doi: 10.1371/journal.pone.0102614 (PMC4085029; doi:10.1371/journal.pone.0102614)
Supplement: File S1 — Originally published, uncorrected article (PDF) [file pone.0102614.s001.pdf]

OPEN ACCESS Freely available online

PLOS | ONE

Subunits of the *Drosophila* Actin-Capping Protein Heterodimer Regulate Each Other at Multiple Levels

Ana Rita Amândio, Pedro Gaspar, Jessica L. Whited¤, Florence Janody*

Instituto Gulbenkian de Ciência, Oeiras, Portugal

Abstract

The actin-Capping Protein heterodimer, composed of the α and β subunits, is a master F-actin regulator. In addition to its role in many cellular processes, Capping Protein acts as a main tumor suppressor module in *Drosophila* and in humans, in part, by restricting the activity of Yorkie/YAP/TAZ oncogenes. We aimed in this report to understand how both subunits regulate each other *in vivo*. We show that the levels and capping activities of both subunits must be tightly regulated to control F-actin levels and consequently growth of the *Drosophila* wing. Overexpressing *capping protein* α and β decreases both F-actin levels and tissue growth, while expressing forms of Capping Protein that have dominant negative effects on F-actin promote tissue growth. Both subunits regulate each other's protein levels. In addition, overexpressing one of the subunit in tissues knocked-down for the other increases the mRNA and protein levels of the subunit knocked-down and compensates for its loss. We propose that the ability of the α and β subunits to control each other's levels assures that a pool of functional heterodimer is produced in sufficient quantities to restrict the development of tumor but not in excess to sustain normal tissue growth.

Citation: Amândio AR, Gaspar P, Whited JL, Janody F (2014) Subunits of the *Drosophila* Actin-Capping Protein Heterodimer Regulate Each Other at Multiple Levels. PLoS ONE 9(5): e96326. doi:10.1371/journal.pone.0096326

Editor: Franck Pichaud, MRC, University College of London, United Kingdom

Received January 21, 2014; **Accepted** April 7, 2014; **Published** May 2, 2014

Copyright: © 2014 Amândio et al. This s an open-access article distributed under the terms of the Creative Commons Attribution License, which permits unrestricted use, distribution, and reproduction n any medium, provided the original author and source are credited.

Funding: This work was supported by grants from Fundação para a Ciência e Tecnologia (FCT) (Grants PTDC/BIA-BCM/121455/2010). Pedro Gaspar was the recipients of fellowships from FCT SFRH/BD/47261/2008. The funders had no role n study design, data collection and analysis, decision to publish, or preparation of the manuscript.

Competing Interests: The authors have declared that no competing nterests exist.

* E-mail: fjanody@igc.gulbenkian.pt

¤ Current address: Regenerative Medicine Center and Department of Orthopedics, Brigham & Women's Hospital, Harvard Medical School, Cambridge, Massachusetts, United States of America

Introduction

The actin cytoskeleton controls numerous processes, ncluding cell shape, mobility, division and ntracellular transport. n normal cells, the actin cytoskeleton s tightly controlled to regulate these essential functions; however, t can be subverted by cancer cells and contributes to changes n cell growth, proliferation, stiffness, movement and nvasiveness [1,2]. Moreover, alterations n the activity or expression of actin-binding proteins (ABPs) *per se*, have been linked to cancer nitiation and progression [2,3,4,5,6].

Among these actin regulators, the actin Capping Protein (CP) heterodimer, composed of an α and a β subunit, appears to act as a main tumor suppressor module [7,8,9,10]. CP was named based on ts ability to bind and cap actin filament barbed ends, nhibiting the addition and loss of actin monomers [11,12,13]. CP has homologs n nearly all eukaryotic cells, ncluding vertebrates, nvertebrates, plants, fungi, nsects and protozoa [14]. *Drosophila* and organisms other than vertebrates have single genes encoding *capping protein* α *(cpa)* or β *(cpb)*. n contrast, vertebrates contain two genes expressed somatically that encode two α subunits (α1 and α2), and one single gene that produce two β soforms (β1 and β2) through alternative splicing [15,16,17]. Although the amino acid sequences of the α and β subunits are not more similar to each other than they are to other ABPs, nor they share common sequences with other proteins, they have extremely similar secondary and tertiary structures [18]. When n complex, the heterodimer resembles a mushroom with the C-terminus of each subunit forming tentacles located on the top surface of the heterodimer [19,20]. *n vitro* analyses of chicken and budding yeast CP revealed that deletions or point mutations n either the α or β tentacles do not affect protein stability but reduce the capping affinity, while a complete removal of both tentacles fully abrogates the actin-binding activity [12,20]. Thus, CP appears to cap F-actin barbed ends via the ndependent nteraction of both tentacles with actin. *n vivo*, a truncated form of *Drosophila cpa* deleted of the C-terminal 28 amino acids has no effect on F-actin when expressed alone but promotes F-actin accumulation when co-expressed with full length *cpb* [21]. Similarly, a chicken β subunit containing a point mutation changing a conserved leucine to arginine at position 262, which caps actin poorly, disrupts the early steps n myofibrillogenesis of cultured myotubes and the sarcomere of mouse heart [22,23,24].

In yeast and *Drosophila*, removing either *cpa* or *cpb* nduces F-actin accumulation and dentical phenotypes [25,26,27]. n the fly, CP s required for proper differentiation of adult bristles, survival of the adult retina, determination of the oocyte and cortical ntegrity of nurse cells n the egg chamber [27,28,29,30]. n addition, CP has a key role n restricting tissue growth. n the whole wing disc epithelium, CP-dependent F-actin regulation suppresses nappropriate tissue growth by nhibiting the activity of the Yorkie (Yki) oncogene, which mediates Hippo signalling activity [7,9]. This function s conserved, as the α1 subunit s also required to limit the activity of the Yki orthologs YAP and TAZ n mammary epithelial cells [31]. n addition, n the distal *Drosophila* wing disc

epithelium, CP prevents JNK-mediated apoptosis or proliferation and counteracts the oncogenic ability of Src [8,21,32]. Furthermore, underexpression of the human α1 subunit correlates with cancer-related death and causes a significant ncrease n gastric cancer cell migration and nvasion *n vitro*, whereas ts overexpression has the opposite effect [10].

We aimed n this report to understand how both subunits regulate each other *n vivo* to control F-actin levels and tissue growth. We show that Cpa and Cpb stabilize each other's protein levels and can stimulate the production of each other's mRNA when the level of one of the subunit s reduced. Because overexpressing *CP* decreases F-actin levels and tissue growth, while expressing forms of CP mutated n their actin-binding domains has opposite effects, we propose that by regulating each other, Cpa and Cpb assure that a pool of functional CP heterodimer s produced n sufficient quantities to restrict tissue growth and therein prevent tumor development but not n excess to sustain proper tissue growth.

Materials and Methods

Molecular Biology

To generate *UAS-cpb*^{L262R}, site-directed mutagenesis was performed on the plasmid UAS-*cpb*, using the QuikChange kit (Stratagene, # 200519). The mutated plasmid was confirmed by sequencing and transgenic flies were generated by standard methods.

Fly strains and genetics

Fly stocks used were *sd*-Gal4 [33]; *nub*-Gal4 [34]; *hh*-Gal4 (a gift from T. Tabata); *da*-Gal4 [35]; UAS-*cpa-IR*^{C10}, *UAS-cpa-IR*^{B4} [7]; UAS-*HA-cpa*^{89E}, UAS-*HA-cpa*$^{\Delta ABD}$ [21]; UAS-cpb7 [36]; UAS-*cpb-RI*^{45668} (Vienna *Drosophila* Research Center, VDRC); *cpa*^{107E} [25], *cpb*^{M143} (FlyBase). To generate *cpa* mutant clones marked by the absence of GFP and expressing or not UAS-*HA-cpa*^{89E} or UAS-*HA-cpa*$^{\Delta ABD}$ or UAS-*cpb*^{7}, *w*; FRT42D, *cpa*^{69E}/CyO or *w*; FRT42D, *cpa*^{107E}/CyO; UAS-*HA-cpa*^{89E}/Tm6β or *w*; FRT42D, *cpa*^{107E}/CyO; UAS-*HA-cpa*$^{\Delta ABD}$/Tm6β or *w*; FRT42D, *cpa*^{107E}, UAS-*cpb*^{7}/CyO males were crossed to *y, w,* FRT42D, *ubi*-GFP; *T155*-Gal4, UAS-*flp*/ST females. To generate *cpb* mutant clones marked by the absence of GFP, *w, y*; FRT40A, *cpb*^{M143}/CyOy^{+} males were crossed to *y, w, hs*FLP122; FRT40A, *ubi*-GFP females and the progeny was heat-shocked at first and second nstar larvae. All crosses were maintained at 25°C and the progeny was dissected at end of third nstar larvae.

Antibody Generation

The rabbit anti-Cpa and rabbit anti-Cpb polyclonal antibodies were generated by Metabion nternational AG using full length Cpa or Cpb tagged with Histidine.

Immunohistochemistry and quantification

We performed mmunocytochemistry using the procedure described n Lee and Treisman [37]. Primary antibodies used were mouse anti-Arm (N2 7A1, Developmental Studies Hybridoma Bank (DSHB); 1:10), rat anti-DE-Cad (1:50, CAD2, DSHB), rabbit anti-Cpa (1:200); rabbit anti-Cpb (1:200); mouse anti-HA (Covance 11 MMS101P; 1:1000) and rabbit anti-Caspase 3 (Cell Signalling #9661; 1:50). Rhodamine conjugated phalloidin (Sigma) was used at a concentration of 0.3 μM. Secondary antibodies were from Jackson mmunoresearch, used at 1:200. Wing discs were mounted n VECTASHIELD Mounting Media (Vector Laboratories, nc. #H-1000). Fluorescence mages were obtained on a Leica SP5 confocal microscope or on a LSM 510 Zeiss confocal microscope. The NIH mage J program was used to perform measurements. Quantifications of the ntensity of Caspase 3 signals were performed as described n [21]. Quantifications of the ratio of Phalloidin signal between posterior and anterior wing compartments were performed as described n [7]. To quantify the ratio of Cpa or Cpb signals between the anterior and posterior wing disc compartments, a region of nterest (ROI) of 100 per 50 pixels was selected. The sum of the gray values was measured for each ROI, applied to each compartments for each disc on optical cross sections through distal wing disc epithelium comprising the apical surface. To measure wing size, wing were dissected one to two days after eclosion and maged using the Hamamatsu Orca-ER camera attached to a Zeiss' Stereo Lumar V12 stereoscope. The total area of each wing was outlined and measured using the *area measurement* function. Statistical significance was calculated using a two-tailed *t*-test.

Western Blotting

For each genetic background, proteins were extracted from either four wing maginal discs or four dechorionated embryos using a 2×SDS sample buffer (Sigma #S3401). Samples were frozen n liquid nitrogen, boiled for 5 minutes n 5 μl Sample Buffer 2x, spun at 13,000 g for 1 minute, loaded on a 10% SDS-PAGE gel and transferred to a PVDF membrane (Amersham Hybond-P, GE Healthcare). Proteins were visualized by mmunoblotting using rabbit anti-Cpa (1:2500) or rabbit anti-Cpb (1:2500) or mouse anti-HA (Covance 11 MMS101P; 1:1000) or rabbit anti-Histone H3 (Cell Signalling #9715; 1:3000). HRP-conjugated donkey anti-mouse or donkey anti-rabbit secondary antibodies were used at 1:5000 (Jackson mmunoResearch Laboratories, nc.). Blots were developed using Amersha ECL Plus Western Blotting Detection System (GE Healthcare). Densitometric analysis of signal ntensity was performed using the GelQuant.NET software (biochemlabsolutions.com) and normalized with the loading control. Statistical significance was calculated using a *Paired t-test.*

Isolation of RNA and Real-Time qRT-PCR

Total RNAs were extracted from either 10 first nstar larvae or 50 wing maginal discs for each genetic background. Samples were homogenized n RLT buffer treated with DNase (Qiagen) at 4 degree C and total RNAs were solated using the RNeasy mini kit (Qiagen) following manufacturer nstructions. First Strand cDNA Synthesis Kit for RT-PCR (Roche) was used to produce cDNAs from 1 μg of total RNA. To quantify mRNA levels, qPCRs were carried out on reverse-transcribed total mRNA using ntron-exon-specific primers (Table S1), designed using the Primer3 software [38,39], and ensuring that efficiency s at least 90% and restricting primer dimmer formation. Real-time qPCR was performed using PerfeCTa SYBR Green FastMix (Quanta Biosciences) n 384 well skirted PCR microplates (Axygen) sealed with optically clear sealing tape (STARSTEDT) n the Applied Biosystems 7900HT Fast Real-Time PCR System. The relative amount of mRNA for each condition was calculated after normalization to the *RpL32* transcript. Statistical significance was calculated using a *Paired t-test* with significance at $P<0.05$.

Results

Cpa and Cpb stabilize each other's protein levels and accumulate at Adherens Junctions

To understand how Cpa and Cpb are regulated to restrict growth of *Drosophila* epithelia, we generated polyclonal antibodies to each CP subunit. n lysates from embryos expressing UAS-*mCD8-GFP* under the control of the ubiquitous *daughterless*-Gal4

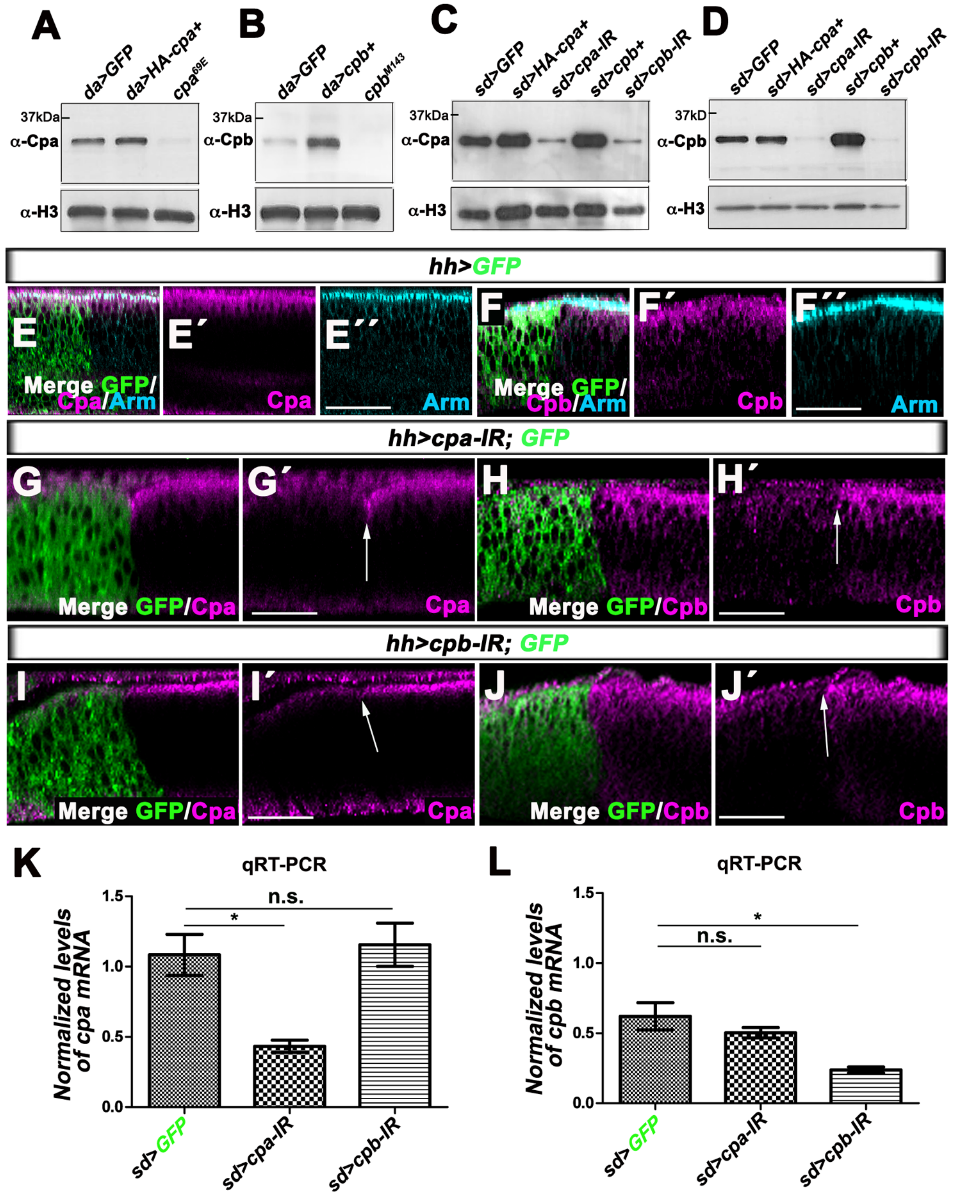

Figure 1. Loss of *cpa* or *cpb* reduces both Cpa and Cpb protein levels. (A) western blot on protein extracts from embryos expressing UAS-*mCD8GFP* (lane 1) or *UAS-HA-*cpa^{89E} (lane 2) under *da*-Gal4 control or homozygote mutant for the cpa^{69E} allele (lane 3), blotted with anti-Cpa (upper panel) and anti-H3 (lower panel). (B) western blot on protein extracts from embryos expressing UAS-*mCD8GFP* (lane 1) or UAS-cpb^{7} (lane 2) under *da*-Gal4 control or homozygote mutant for the cpb^{M143} allele (lane 3), blotted with anti-Cpb (upper panel) and anti-H3 (lower panel). (C and D) western blots on protein extracts from wing maginal discs expressing UAS-*mCD8GFP* (lane 1) or UAS-*HA*-cpa^{89E} (lane 2) or UAS-*cpa*-IR^{C10} (lane 3) or UAS-cpb^{7} (lane 4) or UAS-*cpb*-IR^{45668} (lane 5) under *sd*-Gal4 control, blotted with (C) anti-Cpa (upper panel) and anti-H3 (lower panel) or (D) anti-Cpb (upper panel) and anti-H3 (lower panel). (E-E′′ to J-J′) optical cross sections through distal wing disc epithelium of third nstar larvae with apical side up n which *hh*-Gal4 drives (E–E′′ and F–F′′) UAS-*mCD8-GFP* (green n E and F) and (G–G′ and H–H′) UAS-*cpa*-IR^{C10} or (I–I′ and J–J′) UAS-*cpb*-IR^{45668}. Discs are stained with (E–E′′, G–G′ and -I′) anti-Cpa (magenta) or (F–F′′, H–H′ and J–J′) anti-Cpb (magenta) and (E–E′′ and F–F′′) anti-Arm. The arrows n G′, H′, ′ and J′ mark the limits of the posterior compartment boundary. The scale bars represent 15 μm. (K and L) graphs of (K) *cpa* or (L) *cpb* mRNA levels measured by five ndependent qRT-PCR n wing maginal discs expressing UAS-*mCD8GFP* (lane 1) or UAS-*cpa*-IR^{C10} (lane 2) or UAS-*cpb*-IR^{45668} (lane 3) under *sd*-Gal4 control. (K) the mean for *sd>GFP* s 1.084; for *sd>cpa*-IR^{C10}s 0.4328; for *sd>cpb*-IR^{45668} s 1.155. $P<0.0027$ for comparison of lane 1 and 2. (L) the mean for *sd>GFP* s 0.6210; for *sd>cpa*-IR^{C10} s 0.5037; for *sd>cpb*-IR^{45668} s 0.2375. $P<0.0049$ for comparison of lane 1 and 3. n.s. ndicates a non-significant *P*. Error bars ndicate s.e.m.

doi:10.1371/journal.pone.0096326.g001

(*da*-Gal4) driver, the Cpa (Fig. 1A) and Cpb (Fig. 1B) antibodies revealed a band at around 32 and 31 kDa respectively by Western Blot. These signals were lost n embryonic extracts from homozygous *cpa* (Fig. 1A) or *cpb* mutants (Fig. 1B) respectively. Conversely, overexpressing full length *cpa*, tagged with HA (UAS-*HA-cpa*$^{+}$; Fig. 1A) or *cpb* (UAS-*cpb*$^{+}$; Fig. 1B) with *da*-Gal4, enhanced the anti-Cpa or anti-Cpb signals respectively. Similarly, Cpa levels were ncreased n wing disc lysates overexpressing *HA-cpa* under *scalloped*-Gal4 control (*sd>HA-cpa*$^{+}$; Fig. 1C), while endogenous Cpb levels were similar to control *sd>GFP* lysates

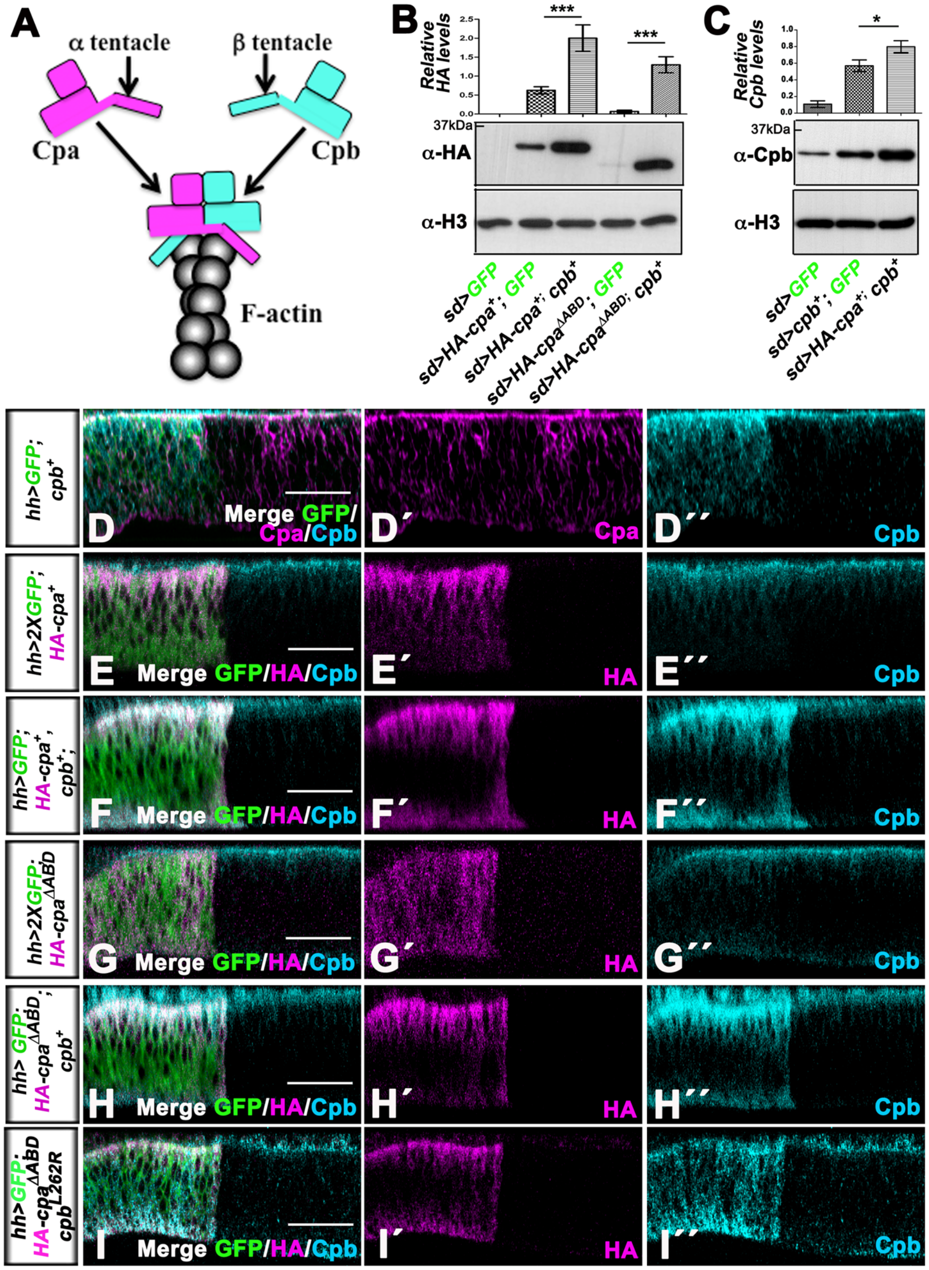

Figure 2. ncreasing the levels of ndividual CP subunits alone has no effect on the endogenous levels of the other subunit, while co-expressing *HA-cpa* or *HA-cpa*$^{\Delta ABD}$ and *cpb* enhance synergistically the levels of both subunits. (A) model by which Cpa and Cpb cap F-actin barbed ends via the nteraction of α and β tentacles with actin. (B) western blot on protein extracts from wing discs expressing UAS-*mCD8-GFP* (lane 1) or UAS-*mCD8-GFP* and UAS-*HA-cpa*^{89E} (lane 2) or UAS-*HA-cpa*^{89E} and UAS-*cpb*^{7} (lane 3) or UAS-*mCD8-GFP* and UAS-*HA-cpa*$^{\Delta ABD}$ (lane 4) or UAS-*HA-cpa*$^{\Delta ABD}$ and UAS-*cpb*^{7} (lane 5) under *sd*-Gal4 control, blotted with anti-HA (middle panel) and anti-H3 (lower panel). The means for lane 1 s 0, for lane 2 s 0.6250, for lane 3 s 2, for lane 4 s 0.0667, for lane 5 s 1.300. Error bars ndicate s.e.m. $P<0.0092$ for comparison of lanes 2 and 3 and of lanes 4 and 5. (B) western blot on protein extracts from wing discs expressing UAS-*mCD8-GFP* (lane 1) or UAS-*mCD8-GFP* and UAS-*cpb*^{7} (lane 2) or UAS-*cpb*^{7} and UAS-*HA-cpa*^{89E} (lane 3) under *sd*-Gal4 control, blotted with anti-Cpb (middle panel) and anti-H3 (lower panel). The upper panels n B and C represent a quantification of relative (B) HA or (C) Cpb ntensity signals for each genetic combination, measured by 4 ndependent blots. The means for lane 1 s 0.1088, for lane 2 s 0.5699, for lane 3 s 0.7982. Error bars ndicate s.e.m. $P<0.0182$ for comparison of lanes 2 and 3. (D–D′′ to -I′′) optical cross sections through distal epithelia of third nstar wing maginal discs with apical sides up and posterior sides to the left n which *hh*-Gal4 drives (D–D′′) UAS-*cpb*^{7} and one copy of UAS-*mCD8-GFP* (green n D) or (E–E′′) UAS-*HA-cpa*^{89E} and two copies of UAS-*mCD8-GFP* (green n E) or (F–F′′) UAS-*HA-cpa*^{89E}, UAS-*cpb*^{7} and one copy of UAS-*mCD8-GFP* (green n F) or (G–G′′) UAS-*HA-cpa*$^{\Delta ABD}$ and two copies of UAS-*mCD8-GFP* (green n G) or (H–H′′) UAS-*HA-cpa*$^{\Delta ABD}$, UAS-*cpb*^{7} and one copy of UAS-*mCD8-GFP* (green n H) or (I-I′′) UAS-*cpb*^{L262R}, UAS-*HA-cpa*$^{\Delta ABD}$ and one copy of UAS-*mCD8-GFP* (green n). Discs are stained with anti-Cpb (Cyan blue) and (D–D′′) anti-Cpa (magenta) or (E–E′′ to -I′′) anti-HA (magenta), which reveals (E–E′′ and F–F′′) *HA-cpa*^{89E} or (G–G′′ to -I′′) *HA-cpa*$^{\Delta ABD}$ expression. The scale bars represent 15 μm.

doi:10.1371/journal.pone.0096326.g002

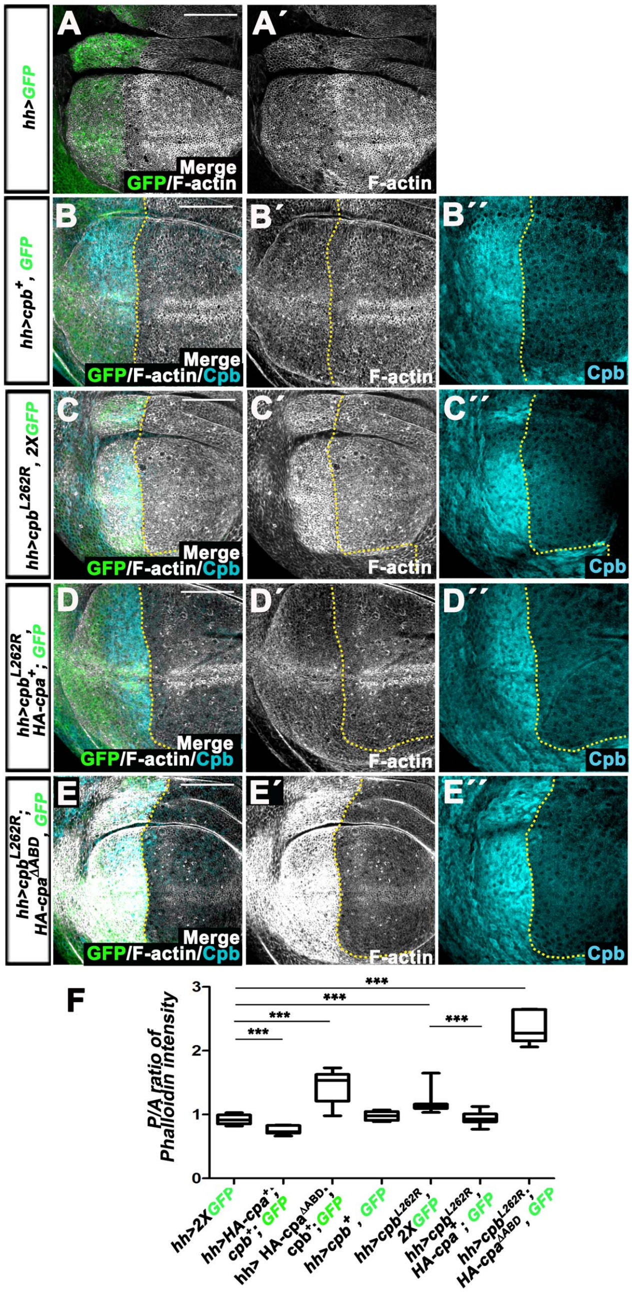

Figure 3. Overexpressing *HA-cpa* suppresses the apical F-actin accumulation of *cpb*^{L262R}-expressing wing discs, whereas *HA-cpa*$^{\Delta ABD}$ expression has the opposite effect. (A-A′′ to E–E′′) standard confocal sections of the apical cell membrane of third nstar wing maginal discs with dorsal sides up and posterior sides to the left, expressing (A–A′) one copy of UAS-*mCD8-GFP* (green n A) or (B–B′′) UAS-*cpb*^{7} and one copy of UAS-*mCD8-GFP* (green n B) or (C–C′′) UAS-*cpb*^{L262R} and two copies of UAS-*mCD8-GFP* (green n C) or (D–D′′) UAS-*cpb*^{L262R}, UAS-*HA-cpa*^{89E} and one copy of UAS-*mCD8-GFP* (green n D) or (E–E′′) UAS-*cpb*^{L262R}, *UAS-HA-cpa*$^{\Delta ABD}$ and one copy of UAS-*mCD8-GFP* (green n E) under *hh*-Gal4 control. Discs are stained with Phalloidin (white) to mark F-actin and (B–B′′ to E–E′′) anti-Cpb (cyan blue). The yellow lines outline the anterior-posterior compartment boundary. The scale bars represent 30 μm. (F) Mean ntensity of the ratio of Phalloidin signal between posterior and anterior wing compartments of *hh*-Gal4 driving two copies of UAS-*mCD8-GFP* (lane 1) or UAS-*HA-cpa*^{89E}, UAS-*cpb*^{7} and one copy of UAS-*mCD8-GFP* (lane 2) or UAS-*HA-cpa*$^{\Delta ABD}$, UAS-*cpb*^{7} and one copy of UAS-*mCD8-GFP* (lane 3) or UAS-*cpb*^{7} and one copy of UAS-*mCD8-GFP* (lane 4) or UAS-*cpb*^{L262R} and two copies of UAS-*mCD8-GFP* (lane 5) or UAS-*cpb*^{L262R}, UAS-*HA-cpa*^{89E} and one copy of UAS-*mCD8-GFP* (lane 6) or UAS-*cpb*^{L262R}, UAS-*HA-cpa*$^{\Delta ABD}$ and one copy of UAS-*mCD8-GFP* (lane 7). The mean for lane 1 s 0.922 (n = 12) for lane 2 s 0.775 (n = 8), for lane 3 s 1.435 (n = 10), for lane 4 s 0.977 (n = 10), for lane 5 s 1.175 (n = 16), for lane 6 s.0.937 (n = 14), for 7 s 2.348 (n = 6). Error bars ndicate s.e.m. *** ndicate $P<0.0001$.
doi:10.1371/journal.pone.0096326.g003

(Fig. 1D). Forcing *cpb* expression n this tissue also nduced a significant ncrease n Cpb levels by Western Blot (Fig. 1D) but did not significantly affect endogenous Cpa levels (Fig. 1C). Cross-sections through wing disc epithelia expressing UAS-*mCD8-GFP* n the posterior compartment using the *hedgehog*-Gal4 (*hh*-Gal4) driver showed that Cpa (Fig. 1E–E′′′) and Cpb (Fig. 1F–F′′′) accumulated at the apical cell membrane and co-localized with components of Adherens Junctions, ncluding the β-Catenin homolog Armadillo (Arm). Co-expressing *cpb* and *mCD8-GFP* n this domain strongly enhanced the anti-Cpb signals but did not affect Cpa levels (Fig. 2D–D′′). Conversely, *hh*>*HA-cpa*$^{+}$ wing disc epithelia displayed an apical localization of HA-Cpa, like endogenous Cpa (Fig. 1E–E′′), but no change n Cpb levels (Fig. 2E–E′′). Thus, the anti-Cpa and Cpb antibodies recognize specifically Cpa and Cpb respectively.

Strikingly, Cpa levels were strongly reduced not only n wing disc extracts expressing double-stranded RNAs (dsRNA) for *cpa* under *sd*-Gal4 control (*sd*>*cpa-IR*) but also n discs knocked-down for *cpb* (*sd*>*cpb-IR*; Fig. 1C). n the converse experiment, the amount of Cpb was also strongly reduced n both *sd*>*cpb-IR* and *sd*>*cpa-IR* wing disc extracts (Fig. 1D). Similarly, knocking down *cpa* (Fig. 1G–G′ and H–H′) or *cpb* (Fig. 1I–I′ and J–J′) n the posterior wing disc compartment with *hh*-Gal4 significantly reduced the apical accumulation of both Cpa and Cpb when compared to anterior compartments used as nternal controls. Moreover, both Cpa and Cpb levels were also strongly reduced n lysates from first nstar larvae homozygote mutant for *cpa* or *cpb* (Fig. S1A) and n clones mutant for *cpa* or *cpb* (Fig. S1B–B′′ to E–E′′). To verify that the *cpa* dsRNA did not affect *cpb* mRNA and *vice versa*, we performed quantitative RT-PCR (qRT-PCR) experiments on wing maginal discs knocked down for *cpa* or *cpb*. As expected, *sd*>*cpa-IR* or *sd*>*cpb-IR* wing discs showed a significant reduction of *cpa* (Fig. 1K, 2.5±0.43 folds) or *cpb* mRNA (Fig. 1L, 2.6±0.41 folds) levels respectively, relative to control *sd*>*GFP*. However, *cpa* mRNA levels were not significantly affected by a reduction n *cpb* (Fig. 1K), nor were *cpb* mRNA levels reduced n wing discs knocked-down for *cpa* (Fig. 1L). Similarly, a reduction n *cpa* or *cpb* levels had no effect on *cpb* or *cpa* mRNA levels, respectively, n first nstar larvae expressing *cpa-IR* or *cpb-IR* under *da*-Gal4 control (Fig. S1F and G). Taken together, we conclude that Cpa and Cpb accumulate at apical cell membrane and enhance each other's protein levels.

Cpa and Cpb levels are rate limited to form a functional heterodimer

The Capping Protein α and β subunits form a functional heterodimer, which caps F-actin barbed ends via the nteraction of the α and β tentacles with actin (Fig. 1A and [11,12,13,20]). To confirm that the stabilization of Cpa and Cpb's protein levels by each other promotes the formation of a functional heterodimer, we first tested f co-expressing *cpb* and *HA-cpa* would enhance the levels of both subunits by comparing the levels of HA-Cpa and Cpb when overexpressed alone or together, ensuring that each genetic combination contained the same number of UAS transgenes. ndeed, by Western Blot (Fig. 2B, $P<0.0092$) and n wing disc epithelia (Fig. 2 compare F–F′′ with E–E′′), HA levels were strongly enhanced when *HA-cpa* was co-expressed with *cpb*. Similarly, the co-expression of *HA-cpa* and *cpb* strongly ncreased

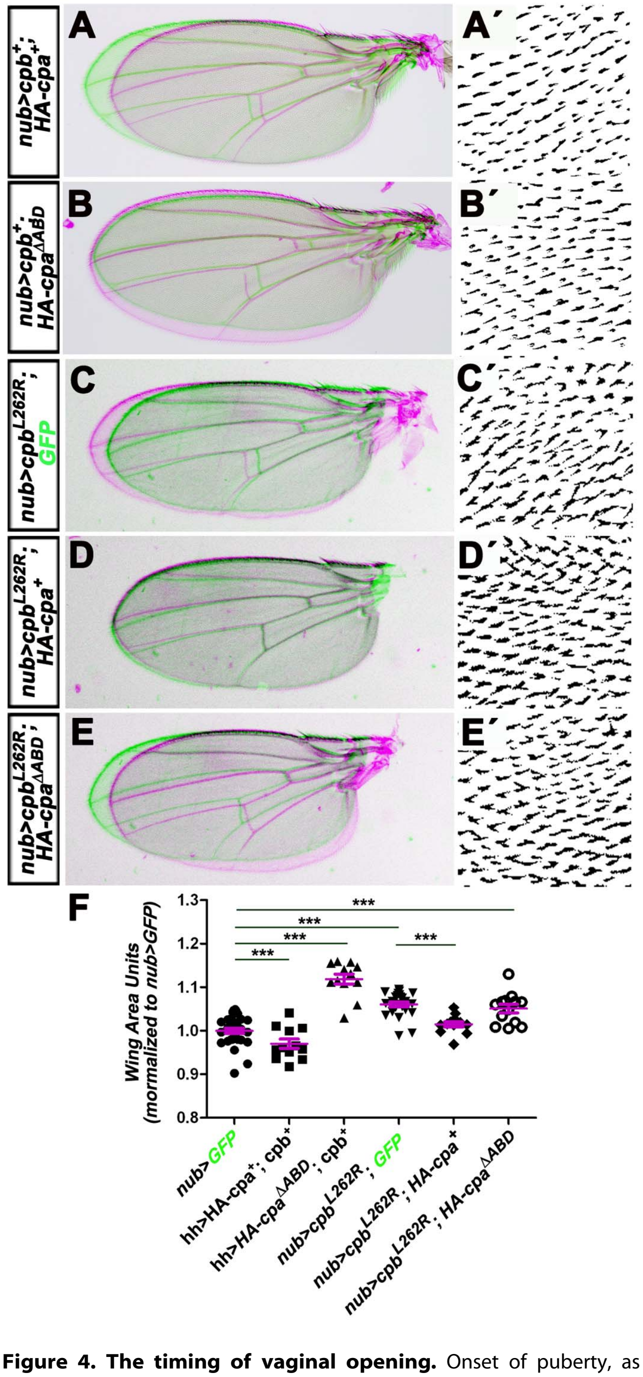

Figure 4. The timing of vaginal opening. Onset of puberty, as marked by the day of vaginal opening, in different groups of rats is presented with respect to age (4A) and body weight (4B). Transplantation of neither fat (CFT), nor ovary (COT) impacts the day of vaginal opening in the control group. But as compared to CFT and COT, the fat transplanted sub-group of the GalTase-exposed rats (GFT) exhibit significantly (P<0.0001) delayed vaginal opening. The timing of vaginal opening, however, is significantly (P<0.0001) advanced in the ovary-transplanted rats of the GalTase-exposed group (GOT). Due to delayed onset of puberty, the body weight on the day of VO is significantly higher in the GFT group with respect to all other groups (P<0.0001). Values are presented as individual datum with mean.
doi:10.1371/journal.pone.0096210.g004

Cpb levels compared to wing disc lysates overexpressing *cpb* alone (Fig. 2C). Overexpressed *HA-cpa* and *cpb* appeared to form a functional heterodimer as their co-expression n the posterior wing disc compartment with *hh*-Gal4 decreased the apical F-actin ratio between both compartments compared to *hh>GFP* control (Fig. 3F, *P<0.0001*). n contrast, overexpressing either *HA-cpa* or *cpb* alone has no effect on F-actin levels [21]. We conclude that the levels of endogenous Cpa and Cpb available are rate limited to form a functional heterodimer.

Forms of CP mutated n α or β tentacle counteract the ability of wild type CP to restrict F-actin accumulation

Surprisingly, expressing an HA-tagged form of Cpa deleted of the α tentacle (UAS-$HA\text{-}cpa^{\Delta ABD}$) has no significant effect on F-actin when expressed alone [21] but triggered apical F-actin accumulation when co-expressed with *cpb* (Fig. 3F, *P<0.0001* and [21]), ndicating that HA-Cpa$^{\Delta ABD}$ affects F-actin only n the presence of overexpressed *cpb*. We therefore tested f the co-expression of *cpb* would also enhance the levels of HA-Cpa$^{\Delta ABD}$. n contrast to full length HA-Cpa, which accumulated apically (Fig. 2E–E″), HA-Cpa$^{\Delta ABD}$ localized uniformly along the apical-basal axis n the posterior compartment of $hh>HA\text{-}cpa^{\Delta ABD}$ wing discs (Fig. 2G–G″). Strikingly, co-expressing *cpb* not only enhanced strongly HA-Cpa$^{\Delta ABD}$ levels as assessed by Western Blot (Fig. 2B, *P<0.0002*), but also relocalized HA-Cpa$^{\Delta ABD}$ at the apical cell membrane (Fig. 2H–H″). Thus, forcing Cpb levels enhances the levels of HA-Cpa$^{\Delta ABD}$ and promotes ts apical localization.

The heterodimer formed between HA-Cpa$^{\Delta ABD}$ and Cpb appears to have reduced capping activity and may be recruited to F-actin barbed ends, preventing the binding of wild type CP. f so, we would expect that a form of Cpb truncated of ts β tentacle would also promote F-actin accumulation n the presence of endogenous CP. To test this possibility, we expressed a form of *cpb* mutated n the highly conserved Leucine 262 (UAS-cpb^{L262R}), which has been proposed to directly nteract with actin [12]. While overexpressing full length *cpb* had no significant effect on F-actin (Fig. 3 compare B–B″ with A–A′ and F), $hh>cpb^{L262R}$ wing discs accumulated apical F-actin n the posterior compartment (Fig. 3C-C″ and F, *P<0.0001*). However, co-expressing full length *HA-cpa* n these tissues suppressed the apical F-actin accumulation due to the presence Cpb^{L262R} (Fig. 3D–D″ and F, *P<0.0001*). Thus, forcing Cpa levels tethers the effects of Cpb^{L262R} on F-actin. n contrast, F-actin accumulation was strongly enhanced when cpb^{L262R} was co-expressed with $HA\text{-}cpa^{\Delta ABD}$ (Fig. 3E-E″ and F, *P<0.0001*). Moreover, Cpb^{L262R}, like full length Cpb, enhances HA-Cpa$^{\Delta ABD}$ levels and triggered ts relocalization to the apical cell membrane (Fig. 2I–I″). We conclude that forms of CP with reduced capping activity nhibit wild type CP to restrict F-actin accumulation, most likely by tethering barbed ends, preventing the recruitment of wild type CP.

CP and forms of CP with dominant negative effects on F-actin have opposite effects on tissue growth

Decreasing or ncreasing CP levels has opposite effects on F-actin levels (Fig. 3F and [25]). Because loss of *CP* nduces overgrowth of the wing disc epithelium by promoting Yki activity [7,9], we asked of overexpressing *cpa* and *cpb* has an opposite effect on tissue growth. ndeed, overexpressing full length *HA-cpa* and *cpb* n the wing primordium using the *nubbin*-Gal4 (*nub*-Gal4) driver significantly reduced the size of the adult wing (Fig. 4A, compare *nub>GFP* control wing n green to $nub>cpa^{+}$, cpb^{+} wing n magenta and F; *P<0.0151*), but does not affect cell survival [21]. Thus, tight CP levels are critical to control tissue growth.

To determine f CP controls tissue growth via F-actin regulation, we analyzed the effect of expressing forms of *cpa* and *cpb* that have

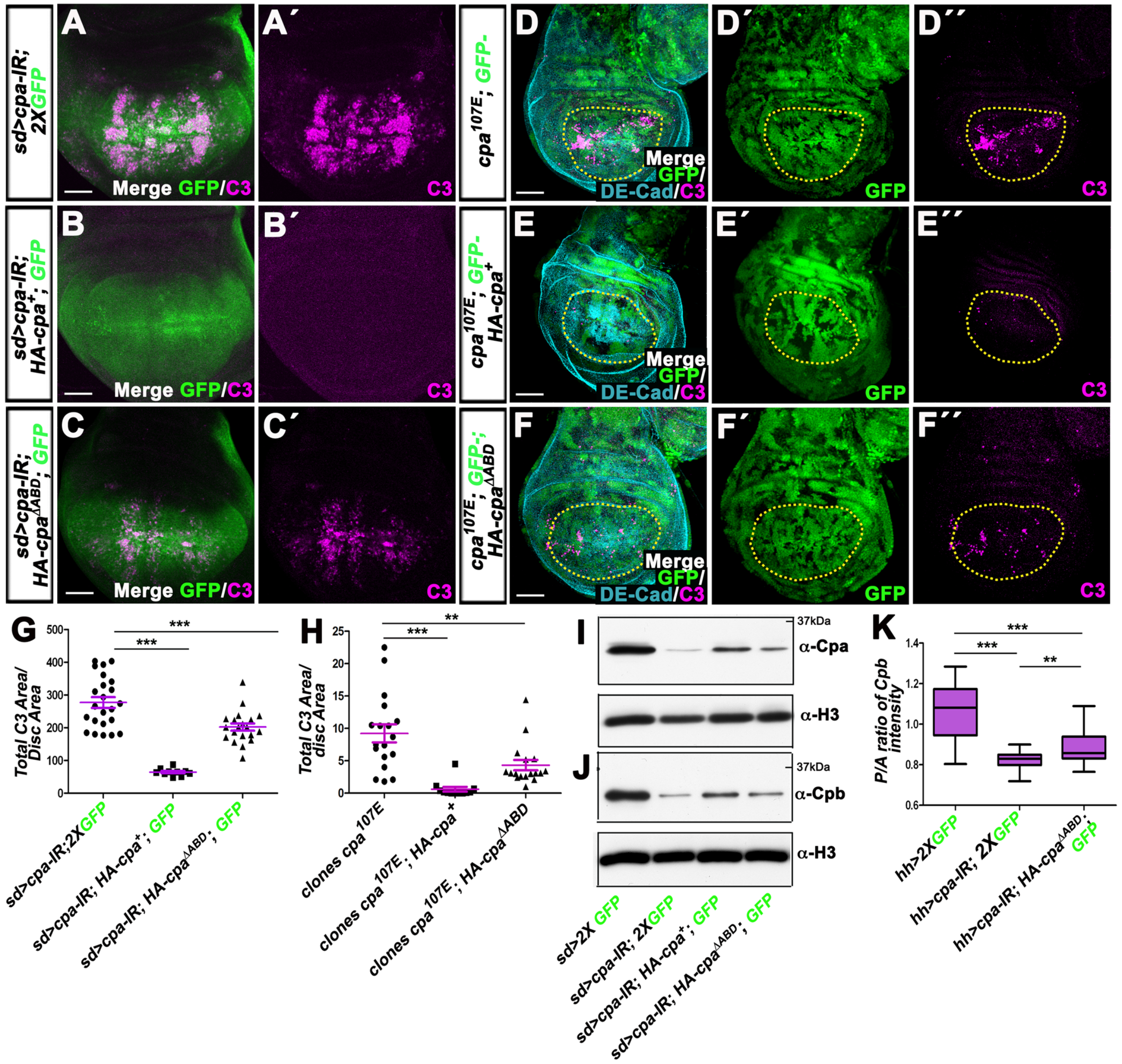

Figure 5. Expressing *HA-cpa* or *HA-cpa$^{\Delta ABD}$* suppresses apoptosis and restores Cpb levels of wing discs knocked-down for *cpa*. (A–A′ to F–F′) standard confocal sections of third nstar wing maginal discs with dorsal sides up. (A–A′ to C–C′) *sd*-Gal4 driving (A–A′) UAS-*cpa-IR^{C10}* and two copies of UAS-*mCD8-GFP* (green n A) or (B–B′) UAS-*cpa-IR^{C10}*, UAS-*HA-cpa^{89E}* and one copy of UAS-*mCD8-GFP* (green n B) or (C–C′) UAS-*cpa-IR^{C10}*, UAS-*HA-cpa$^{\Delta ABD}$* and one copy of UAS-*mCD8-GFP* (green n C). (D–D′′ to F–F′′) *T155*-Gal4; UAS-*flp* nduced *cpa^{107E}* mutant clones marked by the absence of GFP (green) and expressing (E–E′′) UAS-*HA-cpa^{89E}* or (F–F′′) UAS-*HA-cpa$^{\Delta ABD}$* n the whole wing disc epithelium. Discs are stained with anti-activated-Caspase 3 (magenta), which monitors DRONC activation and (D–D′′ to F–F′′) anti-DE-Cad (cyan blue). The scale bars represent 30 µm. (G) quantification of total C3 area per disc area for the three genotypes shown n A–A′ to C–C′. The mean for *sd>cpa-IR^{C10}, 2XGFP* s 92.4 (n = 23); for *sd>cpa-IR^{C10}, HA-cpa^{89E}, 1XGFP* s 0.7 (n = 10); for *sd>cpa-IR^{C10}, HA-cpa$^{\Delta ABD}$, 1XGFP* s 51.4 (n = 20). Error bars ndicate s.e.m. *$P<0.0001$* for comparison of lane 1 and 2. *$P<0.0005$* for comparison of lane 1 and 3. (H) quantification of total C3 area per disc area for the three genotypes shown n D–D′′ to F–F′′. The means for *T155>flp; cpa^{107E}* s 9.228 (n = 18); for *T155>flp; cpa^{107E}*; UAS-*HA-cpa^{89E}* s 0.608 (n = 12); for *T155>flp; cpa^{107E}*; UAS-*HA-cpa$^{\Delta ABD}$* s 4.329 (n = 17). Error bars ndicate s.e.m. *$P<0.0001$* for comparison of *T155>flp; cpa^{107E}* and *T155>flp; cpa^{107E}*; UAS-*HA-cpa^{89E}* and *$P<0.0048$* for comparison of *T155>flp; cpa^{107E}* and *T155>flp; cpa^{107E}*; UAS-*HA-cpa$^{\Delta ABD}$*. (I and J) western blots on protein extracts from wing discs expressing two copies of UAS-*mCD8-GFP* (lane 1) or UAS-*cpa-IR^{C10}* and two copies of UAS-*mCD8-GFP* (lane 2) or UAS-*cpa-IR^{C10}* and UAS-*HA-cpa^{89E}* and one copy of UAS-*mCD8-GFP* (lane 3) or UAS-*cpa-IR^{C10}* and UAS-*HA-cpa$^{\Delta ABD}$* and one copy of UAS-*mCD8-GFP* (lane 4) under *sd*-Gal4 control, blotted with (I) anti-Cpa (upper panel) and anti-H3 (lower panel) or (J) anti-Cpb (upper panel) and anti-H3 (lower panel). (K) mean ntensity of the ratio of Cpb ntensity signals between posterior and anterior wing compartments of *hh*-Gal4 driving two copies of UAS-*mCD8-GFP* (lane 1) or UAS-*cpa-IR^{C10}* and two copies of UAS-*mCD8-GFP* (lane 2) or UAS-*cpa-IR^{C10}* and UAS-*HA-cpa$^{\Delta ABD}$* and one copy of UAS-*mCD8-GFP* (lane 3). The mean for lane 1 s 1.064 (n = 20), for lane 2 s 0.822 (n = 17), for lane 3 s 0.883 (n = 24). Error bars ndicate s.e.m. *$P<0.0001$* for comparison of lanes 1 and 2 or 3 or *$P<0.0085$* for comparison of lanes 2 and 3.

doi:10.1371/journal.pone.0096326.g005

dominant negative effects on F-actin on wing growth. Expressing *HA-cpa$^{\Delta ABD}$* and *cpb* (Fig. 4B and F, *$P<0.0001$*) or *cpb^{L262R}* alone (Fig. 4C and F, *$P<0.0001$*) or combined with *HA-cpa$^{\Delta ABD}$* (Fig. 4E and F, *$P<0.0001$*) under *nub*-Gal4 control, not only promoted apical F-actin accumulation (Fig. 3), but also enhanced significantly the growth of adult wings. Strikingly, expressing *HA-cpa*

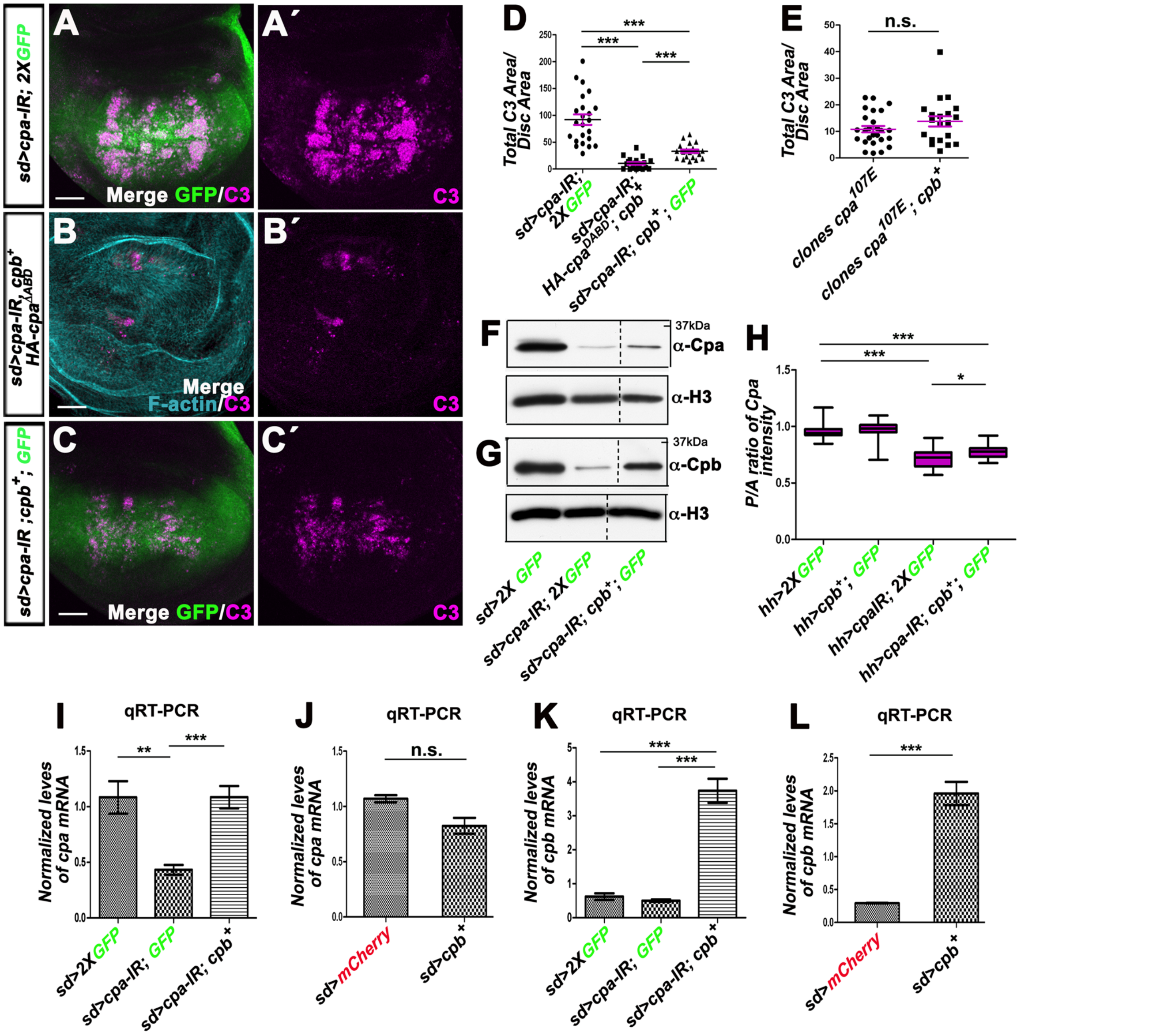

Figure 6. Overexpressing *cpb* n wing discs knocked-down for *cpa*, restores *cpa* mRNA and protein levels and suppresses apoptosis. (A–A′ to C–C′) standard confocal sections of third nstar wing maginal discs with dorsal sides up, expressing (A–A′′) UAS-*cpa-IR*^{C10} and two copies of UAS-*mCD8-GFP* (green n A) or (B–B′) UAS-*cpa-IR*^{C10}, UAS-*HA-cpa*$^{\Delta ABD}$ and UAS-*cpb*^{7} or (C-C′) UAS-*cpa-IR*^{C10}, UAS-*cpb*^{7} and one copy of UAS-*mCD8-GFP* (green n C) under *sd*-Gal4 control. Discs are stained with anti-activated-Caspase 3 (magenta), which monitors DRONC activation and (B-B′) Phalloidin (cyan blue n B) to underline wing disc shape. The scale bars represent 30 µm. (D) quantification of total C3 area per disc area for the genotypes *sd*> *cpa-IR*^{C10}, *2XGFP* (lane 1); *sd*>*cpa-IR*^{C10}, *HA-cpa*$^{\Delta ABD}$, *cpb*^{7} (lane 2) and *sd*>*cpa-IR*^{C10}, *cpb*^{7}, *1XGFP* (lane 3). The means for lane 1 s 92.4 (n = 23); for lane 2 s 10.61 (n = 19); for lane 3 s 32.9 (n = 20). Error bars ndicate s.e.m. $P<0.0001$ for comparison of lane 1 and 2 or 3 or lane 2 and 3. (E) quantification of total C3 area per disc area for wing discs containing *T155*>*flp; cpa*^{107E} mutant clones (lane 1) or *T155*>*flp; cpa*^{107E} mutant clones expressing UAS-*cpb*^{7} (lane 2). The means for lane 1 s 10.80 (n = 26); for lane 2 s 13.77 (n = 20). n.s. ndicates non-significant *P* value. (F and G) western blots on protein extracts from wing discs expressing two copies of UAS-*mCD8-GFP* (lane 1) or UAS-*cpa-IR*^{C10} and two copies of UAS-*mCD8-GFP* (lane 2) or UAS-*cpa-IR*^{C10}, UAS-*cpb*^{7} and one copy of UAS-*mCD8-GFP* (lane 3) under *sd*-Gal4 control, blotted with (F) anti-Cpa (upper panel) and anti-H3 (lower panel) or (G) anti-Cpb (upper panel) and anti-H3 (lower panel). Panels derive from the same experiment shown n Figure 5E and F and blots were processed n parallel (see Figure S2 showing the whole experiment). (H) mean ntensity of the ratio of Cpa signals between posterior and anterior wing compartments of *hh*-Gal4 driving two copies of UAS-*mCD8-GFP* (lane 1) or UAS-*cpb*^{7} and one copy of UAS-*mCD8-GFP* (lane 2) or UAS-*cpa-IR*^{C10} and two copies of UAS-*mCD8-GFP* (lane 3) or UAS-*cpa-IR*^{C10} and UAS-*cpb*^{7} and one copy of UAS-*mCD8-GFP* (lane 4). The mean for lane 1 s 0.959 (n = 15), for lane 2 s 0.970 (n = 20), for lane 3 s 0.716 (n = 21), for lane 4 s 0.776 (n = 20). Error bars ndicate s.e.m. $P<0.0001$ for comparison of lanes 1 and 3 or 4 or $P<0.01$ for comparison of lanes 3 and 4. (I to L) graph of (I and J) *cpa* or (K and L) *cpb* mRNA levels measured by five ndependent qRT-PCR n wing maginal discs expressing (I and K) two copies of UAS-*mCD8GFP* (lane 1) or UAS-*cpa-IR*^{C10} and UAS-*mCD8GFP* (lane 2) or UAS-*cpa-IR*^{C10} and UAS-*cpb*^{7} (lane 3) or (J and L) UAS-*mCherry* (lane 1) or UAS-*cpb*^{7} (lane 2) under *sd*-Gal4 control. (I) the means for lane 1 s 1.084; for lane 2 s 0.4328; for lane 3 s 1.086. $P<0.0027$ for comparison of lane 1 with 2 or $P<0.0003$ for comparison of lane 2 with 3. (J) the means for lane 1 s 1.07; for lane 2 s 0.824. n.s. ndicates non-significant *P* value. (K) the means for lane 1 s 0.621; for lane 2 s 0.5031; for lane 3 s 3.735. $P<0.0001$ for comparison of lane 3 with 1 or 2. (L) the means for lane 1 s 0.292; for lane 2 s 1.961. $P<0.0001$ for comparison of lane 1 and 2. Error bars ndicate s.e.m.
doi:10.1371/journal.pone.0096326.g006

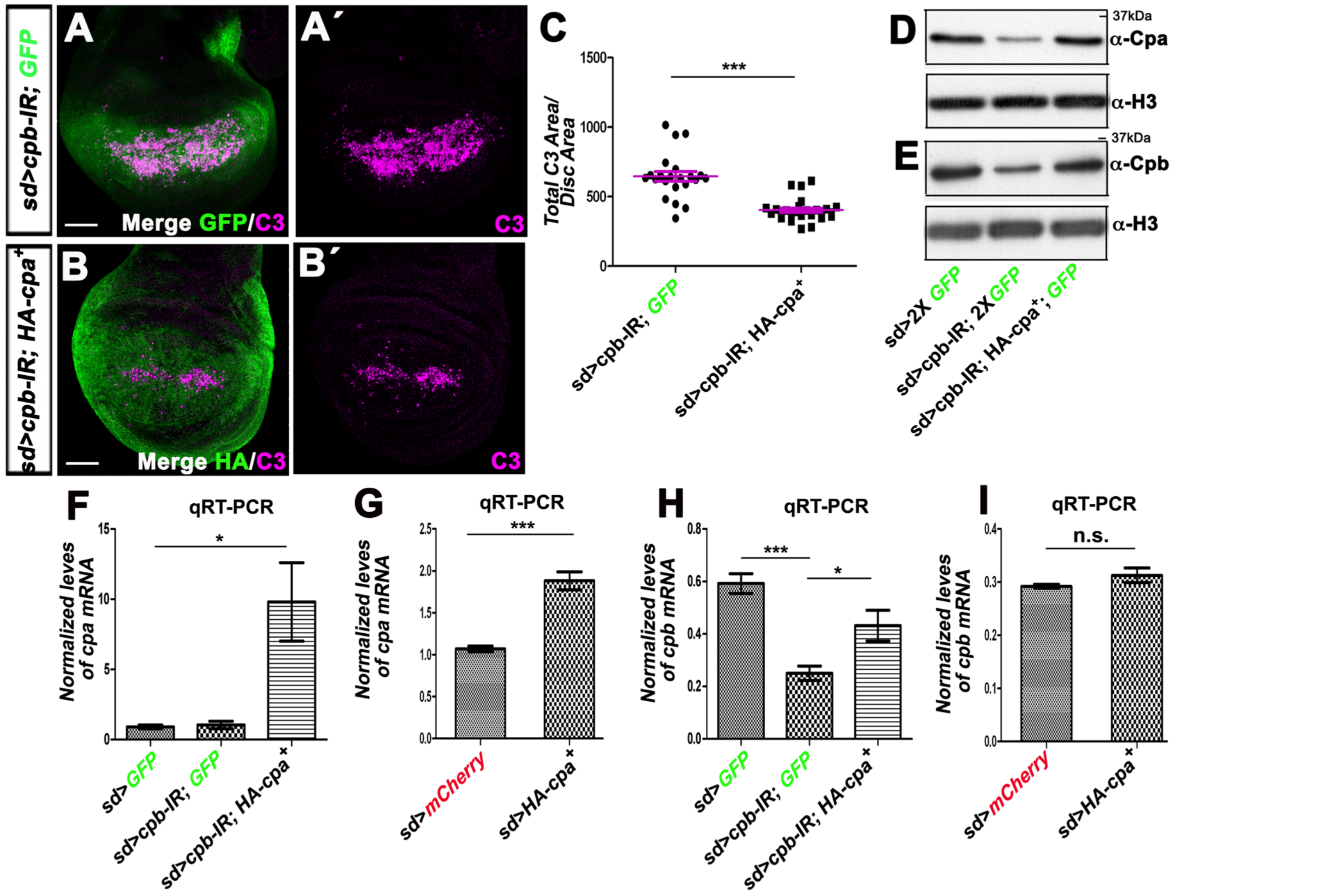

Figure 7. Overexpressing *HA-cpa* n wing discs knocked-down for *cpb* restores *cpb* mRNA and protein levels and suppresses apoptosis. (A–A′ and B–B′) standard confocal sections of third nstar wing maginal discs with dorsal sides up, expressing (A–A′′) UAS-*cpb-IR^{45668}* and one copy of UAS-*mCD8-GFP* (green n A) or (B–B′) UAS-*cpb-IR^{45668}* and UAS-*HA-cpa^{89E}* under *sd*-Gal4 control. Discs are stained with anti-activated-Caspase 3 (magenta), which monitors DRONC activation and (B–B′) anti-HA (green n B), reflecting *HA-cpa^{89E}* expression. The scale bars represent 30 μm. (C) quantification of total C3 area per disc area for the two genotypes shown n A–A′ and B–B′. The means for *sd*>*cpb-IR^{45668}*, *GFP* s 62.19 (n = 22); for *sd*>*cpb-IR^{4566}*, *HA-cpa^{89E}* s 26.67 (n = 32). Error bars ndicate s.e.m. *P*<*0.0001* for comparison of between both genotypes. (D and E) western blots on protein extracts from wing discs expressing two copies of UAS-*mCD8-GFP* (lane 1) or UAS-*cpb-IR^{45668}* and two copies of UAS-*mCD8-GFP* (lane 2) or UAS-*cpb-IR^{45668}* and UAS-HA-*cpa^{89E}* and one copy of UAS-*mCD8-GFP* (lane 3) under *sd*-Gal4 control, blotted with (D) anti-Cpa (upper panel) and anti-H3 (lower panel) or (E) anti-Cpb (upper panel) and anti-H3 (lower panel). (F to) graphs of (F and G) *cpa* or (H and) *cpb* mRNA levels measured by (F and H) three or (G and) five ndependent qRT-PCR n wing maginal discs expressing (F and H) two copies of UAS-*mCD8GFP* (lane 1) or UAS-*cpb-IR^{45668}* and UAS-*mCD8GFP* (lane 2) or UAS-*cpb-IR^{45668}* and UAS-*HA-cpa^{89E}* (lane 3) or (G and) UAS-*mCherry* (lane 1) or UAS-*HA-cpa^{89E}* under *sd*-Gal4 control. (F) the means for lane 1 s 0.90; for lane 2 s 1.04; for lane 3 s 9.8. *P*<*0.033* for comparison of lane 1 with 3. (G) the means for lane s 1.07; for lane 2 s 1.88. *P*<*0.0001* for comparison of lane 1 and 2. (H) the means for lane 1 s 0.59; for lane 2 s 0.25; for lane 3 s 0.4319. *P*<*0.0018* for comparison of lane 1 and 2 or *P*<*0.048* for comparison of lane 2 and 3. (I) the means for lane 1 s 0.29; for lane 2 s 0.31. n.s. ndicates non-significant *P* value. Error bars ndicate s.e.m.

doi:10.1371/journal.pone.0096326.g007

suppressed the overgrowth of *nub*>*cpb^{L262R}* wings (Fig. 4D and F, *P*<*0.0001*), ndicating that the effect of Cpb^{L262R} on F-actin and tissue growth s dependent on the levels of full length Cpa. Because altering the levels or activity of CP did not affect the density of wing hairs (Fig. 4A′, B′, C′ D′ and E′), which develop from one single cell, the CP-dependent growth defects most likely result from changes n proliferation rate rather than alteration of cell size. We conclude that a CP-dependent reduction of F-actin levels correlates with tissue undergrowth, while a CP-dependent ncrease n F-actin levels s associated with tissue overgrowth.

The α tentacle s not absolutely required to form a functional heterodimer

Because the heterodimer formed between HA-Cpa$^{\Delta ABD}$ and Cpb appears to be recruited at F-actin barbed ends, we tested f HA-Cpa$^{\Delta ABD}$ can partially compensate for the loss of endogenous Cpa. Expressing *cpa-IR* under *sd*-Gal4 control nduced the activation of Caspase 3 n numerous cells n the distal wing disc epithelium (Fig. 5A–A′). Apoptosis was almost fully suppressed by overexpressing full length *HA-cpa* (Fig. 5B-B′ and G; *P*<*0.0001*). Expressing *HA-cpa$^{\Delta ABD}$* also significantly prevented apoptosis of *sd*>*cpa-IR* wing discs, although to a much weaker extent than *HA-cpa* (Fig. 5C–C′ and G; *P*<*0.0005*). These effects were not only due to titration of the *cpa* dsRNAs by the overexpressed *cpa* constructs as *HA-cpa* (Fig.5E–E′′ and H) or *HA-cpa$^{\Delta ABD}$* (Fig. 5F–F′′ and H; *P*<*0.0048*) also rescued apoptosis of clones mutant for a *cpa* allele. Expressing *HA-cpa* or *HA-cpa$^{\Delta ABD}$* n *sd*>*cpa-IR* wing discs also partially restored Cpa (Fig. 5I) and Cpb (Fig. 5J) levels, as assessed by Western blot. Quantification of the ratio of Cpb signals between the posterior and anterior compartments of wing discs expressing *cpa-IR* under *hh*-Gal4 control showed that knocking-

down *cpa* reduced Cpb levels n the posterior compartment compared to *hh>GFP* control (Fig. 5K). This decrease n Cpb levels was significantly alleviated by the presence of HA-Cpa$^{\Delta ABD}$ (Fig. 5K *P<0.0085*). We conclude that n the absence of wild type Cpa, Cpa$^{\Delta ABD}$ s capable of forming a functional heterodimer with Cpb, which prevents apoptosis.

Cpb compensates for a reduction n *cpa* by enhancing *cpa* mRNA levels and *vice versa*

Interestingly, co-expressing *cpb* with *HA-cpa*$^{\Delta ABD}$ almost fully suppressed apoptosis of wing discs knocked-down for *cpa* (Fig. 6 compare B–B′ with A–A′ and D; *P<0.0001*). This effect could be due to the stabilization and apical relocalization of HA-Cpa$^{\Delta ABD}$ when co-expressed with *cpb* (Fig. 2H–H″). However, apoptosis of *sd>cpa-IR* wing discs was also significantly suppressed by overexpressing *cpb* alone (Fig. 6C-C′ and D; *P<0.0001*). Conversely, expressing *HA-cpa* n tissues knocked-down for *cpb* (*sd>cpb-IR*) also prevented apoptosis (Fig. 7 compare B–B′ with A–A′ and C; *P<0.0001*).

To understand the mechanisms by which Cpa and Cpb compensate for each other's function, we tested the effect of overexpressing *cpb* on Cpa levels n *cpa*-depleted tissues. As previously observed, by Western Blots, Cpa (Fig. 6F) and Cpb (Fig. 6G) levels were strongly reduced n wing disc extracts knocked-down for *cpa*. Forcing *cpb* levels n these tissues enhanced the levels of both Cpa (Fig. 6F and Fig. S2) and Cpb (Fig. 6G and Fig. S2). We quantified this effect by measuring the ratio of Cpa signals between the posterior and anterior compartments of *hh>cpa-IR*-expressing wing discs, n the presence or absence of overexpressed *cpb*. While n control *hh>GFP* tissues this ratio was 0.95, knocking down *cpa* reduced this ratio to 1,34 folds (Fig. 6H; *P<0.0001*). This effect was significantly alleviated by the overexpression of *cpb* (Fig. 6H*; P<0.01*). n contrast, overexpressing *cpb* n control *hh>GFP* wing discs did not affect Cpa levels (Fig. 6H), ndicating that Cpb enhances Cpa levels only when cells contain reduced Cpa levels. By Western Blots, *HA-cpa* also enhanced both Cpa (Fig. 7D) and Cpb (Fig. 7E) levels when expressed n tissues knocked-down for *cpb*. Thus, Cpa compensates for a reduction n *cpb* by stimulating the production of Cpb, and *vice versa*.

Using qRT-PCR, we next analyzed f overexpressing either subunits affects the mRNA levels of the other. After normalization to the *RpL32* transcript used as an nternal control, we observed that whereas *cpa* (Fig. 6I, *P<0.0027*) but not *cpb* (Fig. 6K) mRNA levels were strongly reduced n wing discs knocked-down for *cpa* (*sd>cpa-IR*), forcing *cpb* levels n these tissues fully restored *cpa* mRNA to wild type levels (Fig. 6I; *P<0.0003*). n contrast, n wing discs that contained endogenous *cpa* and *cpb*, overexpressing *cpb*, which strongly enhanced *cpb* mRNA levels (Fig. 6L), had no significant effect on *cpa* mRNA levels (Fig. 6J). Thus, Cpb stimulates the production or stabilization of *cpa* mRNA only when Cpa levels are reduced. n the converse experiment, overexpressing *HA-cpa* n *sd>cpb*-depleted wing discs enhanced the levels of both *cpa* (Fig. 7F) and *cpb* (Fig. 7H; *P<0.0018*) mRNA. However, n wing discs that contained endogenous *cpa* and *cpb*, only *cpa* mRNA levels were strongly ncreased (Fig. 7G and). The ability of Cpb to suppress apoptosis of *cpa*-depleted wing discs was due to the ncrease n *cpa* mRNA and protein levels as clones mutant for a *cpa* allele showed similar apoptotic levels n the absence or presence of overexpressing *cpb* (Fig. 6E). We conclude that Cpa compensates for a reduction n *cpb* by ncreasing *cpb* mRNA levels and *vice versa*.

Discussion

Cpa and Cpb regulate each other at multiple levels

Our data argue that n *Drosophila*, different pools of Cpa and/or Cpb co-exist, and they regulate each other at various levels. One level of regulation nvolves their reciprocal stabilization of their protein levels. First, n *Drosophila*, like n yeast, the loss of one CP subunit reduces the protein levels of the other subunit ([26] and Fig. 1) but does not affect ts mRNA levels (Fig. 1 and Fig. S1). Second, co-expressing *cpa* and *cpb* n *Drosophila* tissues enhances synergistically the levels of both subunits relative to the levels of each subunit overexpressed alone (Fig. 2). Third, large quantities of soluble active chicken CP can be produced n bacteria only when both subunits are co-expressed [40]. Cpa and Cpb may stabilize each other's protein levels via direct protein-protein nteractions [19]. The tight nteraction between both subunits may prevent the recruitment of E3 ubiquitin ligases that would otherwise target ndividual CP subunits for degradation by the 26 S proteasome. As an heterodimer, CP has been shown to bind to the fast polymerizing ends of actin filaments, preventing further addition of actin monomers [41,42] and to restrict F-actin accumulation n *Drosophila* tissues [25,27]. n addition, Cpa and Cpb appear to show some function on their own as overexpressing *cpb* rescues apoptosis of wing discs knocked-down for *cpa* and *vice versa* (Fig. 6 and 7). Overexpression of *cpb* alone s also sufficient to enhance the retinal defects of flies knocked down for the Cbl-interacting protein *cindr* [43] and to rescue the migration and F-actin polarization defects of *Drosophila* border cells mutant for *warts* [44]. Because ndividual chicken CP subunits expressed n bacteria are mainly deposited nto nsoluble cytoplasmic nclusion bodies but can be renaturated as active heterodimers [45], ndividual subunit may exist n the cell as pools of nsoluble monomers. The molecular mechanism by which ndividual CP subunit compensates for each other's function remains to be determined. Several observations argue that this mechanism nvolves the production of the subunit knocked-down by the other subunit via an ncrease of ts mRNA levels (Fig. 6 and 7). CP has been observed n the nuclei of chicken retinal and kidney epithelial cells n culture, n Madin-Darby canine kidney (MDCK) cells, n *Xenopus laevis* oocytes and bovine lens epithelial cells n culture [46,47]. Whether Cpa and Cpb nfluence each other's transcription n the nucleus s an nteresting possibility to be tested. The protein-mRNA feedbacks between Cpa and Cpb may guarantee that a pool of functional heterodimer s present to limit F-actin polymerization. However, a CP-dependent negative feedback mechanism must exist that restricts the production of CP n excess, as forcing the expression of one of the subunit n tissues that contain endogenous CP does not enhance the mRNA and protein levels of the other subunit (Fig. 6 and 7). Because the loss of one subunit has no effect on the mRNA levels of the other subunit (Fig. 1 and Fig. S1), the CP-dependent negative feedback may act by limiting the ability of ndividual subunits to stimulate the production of each other's mRNAs. Thus, n addition to regulate each other's protein levels, ndividual CP subunit stimulates each other's mRNA production up to an optimal physiological threshold of functional heterodimers. Further experiments are necessary to elucidate the protein-mRNA feedback loop mechanisms, which operate between both subunits.

Capping activity of the CP heterodimer at actin filament barbed ends

Our observations argue that *n vivo* the actin-binding domain of Cpa s not absolutely required to form a functional CP heterodimer, as HA-Cpa$^{\Delta ABD}$ partially compensates for the loss of endogenous Cpa (Fig. 5). Consistent with our observations, n

actin assembly assays, a mutant form of the chicken α subunit that lacks the α tentacle s able to cap F-actin [12]. Nevertheless, the α tentacle may favor the nteraction and therefore stabilization of the α subunit by the β. This possibility s consistent with the observation that HA-Cpa$^{\Delta ABD}$ s found n the cell at much lower levels than full length HA-Cpa (Fig. 2) despite both transgenes being nserted at the same locus n the fly genome and therefore likely expressed at similar levels [21]. Consistent with this hypothesis, Arginine 259 of the chicken α1 tentacle forms side-chain hydrogen bonds with three residues of the β subunit, all residues being conserved across soforms and species [19]. Moreover, *n vitro*, a truncated form of the chicken α1 subunit, consisting only of the C-terminal domain, retains the ability to form a heterodimer [48]. The reduced ability of HA-Cpa$^{\Delta ABD}$ to nteract with Cpb may explain ts nability to fully suppress apoptosis of Cpa-depleted tissues (Fig. 5) and to affect F-actin levels when overexpressed alone [21]. However, several observations ndicate that the α and β tentacles also enable full capping activity *n vivo*. First, n actin assembly assays, the C-terminus of the chicken α1 and β1 subunits are required for high-affinity capping [12]. Second, n the presence of endogenous CP, stabilizing HA-Cpa$^{\Delta ABD}$ levels by forcing *cpb* expression does not reduce F-actin levels, as does overexpressed *HA-cpa/cpb*, but nstead, promotes F-actin accumulation (Fig. 3 and [21]). Third, replacing leucine 262 of the chicken β subunit has no effect on protein stability and global structure but decreases the capping affinity significantly [12,20]. Fourth, dentical mutations n the β orthologs nduces F-actin accumulation n *Drosophila* tissues (Fig. 3) and disrupts the sarcomere of mouse heart [24]. Thus, we propose that the heterodimers formed between HA-Cpa$^{\Delta ABD}$ and Cpb or between Cpb^{L262R} and Cpa are recruited to F-actin barbed ends and cap actin filaments less efficiently than wild type CP. The low capping activity of the HA-Cpa$^{\Delta ABD}$/Cpb heterodimer s sufficient to partially compensate for the loss of Cpa. However, n the presence of endogenous CP, the HA-Cpa$^{\Delta ABD}$/Cpb heterodimers compete with wild type Cpa/Cpb heterodimers for binding the barbed ends of F-actin, which can lead to defects n F-actin.

Tight regulation of CP levels s critical to control tissue growth

CP appears to act as a gatekeeper, which limits the development of cancer-related processes. Loss of the α subunit promotes Yki/YAP/TAZ-dependent proliferation n *Drosophila* epithelia and n human cells [9,31], causes a significantly ncrease n gastric cancer cell migration and s associated with cancer-related death [10]. n contrast, ncreasing CP levels has opposite effects: t reduces tissue growth (Fig. 4) and prevents Src-mediated tumour development n *Drosophila* [21], and significantly restricts gastric cancer cell migration [10]. Several of our observations argue that the function of CP on tissue growth nvolves ts F-actin capping activity. First expressing *cpb*^{L262R}, which contains a single point mutation affecting the capping activity [23], nduces F-actin accumulation (Fig. 3) and wing overgrowth (Fig. 4). Moreover, CP-dependent F-actin accumulation correlates with tissue overgrowth, whereas tissue undergrowth s associated with a CP-dependent reduction n F-actin (Fig. 3 and 4). Consistent with these observations, other actin regulators have been shown to control Yki/YAP/TAZ dependent tissue growth [7,9,31]. Thus, a reduction or an ncrease of CP levels has deleterious consequences on tissue growth, mplying that t must be tightly regulated. This may be achieved n part by the ability of Cpa and Cpb to stimulate or limit the production of each other n conditions of lower or higher CP levels respectively, assuring that a pool of functional CP heterodimer s produced n sufficient quantities n the cell to prevent cancer development but not n excess to sustain proper tissue growth.

Supporting Information

Figure S1 Reducing *cpa* or *cpb* levels reduces both Cpa and Cpb protein levels. (A) western blot on protein extracts from first nstar larvae, either *white minus* (lane 1) or homozygote mutant for *cpa*^{69E} (lane 2) or homozygote mutant for *cpb*^{M143} (lane 3), blotted with (upper panel) anti-Cpa (upper bands) and anti-Cpb (lower band) and (lower panel) anti-H3. (B–B′′ to E–E′′) standard confocal sections of third nstar wing maginal discs, containing (B–B′′ and C–C′′) *T155*-Gal4; UAS-*flp* nduced *cpa*^{69E} mutant clones marked by the absence of GFP (green) or (D–D′′ and E–E′′) heat shocked-induced *cpb*^{M143} mutant clones marked by the absence of GFP (green). Discs are stained with (B–B′′ and E–E′′) anti-Cpa (magenta) or (C–C′′ and E–E′′) anti-Cpb (magenta). The scale bars represent 15 μm. (F and G) graphs of (F) *cpa* or (G) *cpb* mRNA levels measured by three ndependent qRT-PCR n first nstar larvae expressing UAS-*mCD8-GFP* (lane 1) or UAS-*cpa-IR*^{C10} (lane 2) or UAS-*cpb*-IR^{45668} (lane 3) under *da*-Gal4 control. (F) The means for lane 1 s 7.04; for lane 2 s 1.13; for lane 3 s 5.91. Error bars ndicate s.e.m. $P<0.015$ for comparison of lane 1 and 2. (F) The means for lane 1 s 1.97; for lane 2 s 1.96; for lane 3 s 0.46. Error bars ndicate s.e.m. $P<0.021$ for comparison of lane 1 and 3. n.s. ndicates non-significant *P* values.
(TIF)

Figure S2 Expressing *HA-cpa* or *HA-cpa*$^{\Delta ABD}$ or *cpb* n wing discs knocked down for *cpa* restores Cpa and Cpb levels. Western blots on protein extracts from wing discs expressing two copies of UAS-*mCD8-GFP* (lane 1) or UAS-*cpa-IR*^{C10} and two copies of UAS-*mCD8-GFP* (lane 2) or UAS-*cpa-IR*^{C10} and UAS-*HA-cpa*^{89E} and one copy of UAS-*mCD8-GFP* (lane 3) or UAS-*cpa-IR*^{C10} and UAS-*HA-cpa*$^{\Delta ABD}$ and one copy of UAS-*mCD8-GFP* (lane 4) or UAS-*cpa-IR*^{C10} and UAS-*HA-cpa*^{ABD}, which contains the last 28 amino acids of the Cpa C-terminus and one copy of UAS-*mCD8-GFP* (lane 5) or UAS-*cpa-IR*^{C10}, UAS-*cpb*^{7} and one copy of UAS-*mCD8-GFP* (lane 6) under *sd*-Gal4 control, blotted with (A) anti-Cpa (upper panel) and anti-H3 (lower panel) or (B) anti-Cpb (upper panel) and anti-H3 (lower panel).
(TIF)

Table S1 Intron-exon-specific primers used to quantify *cpa*, *cpb* and *RpL32* mRNA levels by qRT-PCR.
(DOCX)

Acknowledgments

We thank the Bloomington *Drosophila* Stock Center, the National nstitute of Genetics and the Developmental Studies Hybridoma Bank for fly stocks. We are grateful to Barbara Jezowska, Beatriz García Fernández, Francesca Vasconcelos, Sonia Rosa, Vania Neves and Claire Brombey for technical support and advice on qRT-PCR. The manuscript was mproved by the critical comments of Claudine Chaouiya and Alekos Athanasiadis.

Author Contributions

Conceived and designed the experiments: ARA PG JLW FJ. Performed the experiments: ARA PG JLW FJ. Analyzed the data: ARA PG FJ. Wrote the paper: FJ.

References

1. Lambrechts A, Van Troys M, Ampe C (2004) The actin cytoskeleton n normal and pathological cell motility. nt J Biochem Cell Biol 36: 1890–1909.
2. Stevenson RP, Veltman D, Machesky LM (2012) Actin-bundling proteins n cancer progression at a glance. J Cell Sci 125: 1073–1079.
3. Shah V, Braverman R, Prasad GL (1998) Suppression of neoplastic transformation and regulation of cytoskeleton by tropomyosins. Somat Cell Mol Genet 24: 273–280.
4. Mahadev K, Raval G, Bharadwaj S, Willingham MC, Lange EM, et al. (2002) Suppression of the transformed phenotype of breast cancer by tropomyosin-1. Exp Cell Res 279: 40–51.
5. Mammoto A, ngber DE (2009) Cytoskeletal control of growth and cell fate switching. Curr Opin Cell Biol 21: 864–870.
6. Yamamoto N, Okano T, Ma X, Adelstein RS, Kelley MW (2009) Myosin regulates extension, growth and patterning n the mammalian cochlear duct. Development 136: 1977–1986.
7. Fernandez BG, Gaspar P, Bras-Pereira C, Jezowska B, Rebelo SR, et al. (2011) Actin-Capping Protein and the Hippo pathway regulate F-actin and tissue growth n Drosophila. Development 138: 2337–2346.
8. Jezowska B, Fernandez BG, Amandio AR, Duarte P, Mendes C, et al. (2011) A dual function of Drosophila capping protein on DE-cadherin maintains epithelial ntegrity and prevents JNK-mediated apoptosis. Dev Biol 360: 143–159.
9. Sansores-Garcia L, Bossuyt W, Wada K, Yonemura S, Tao C, et al. (2011) Modulating F-actin organization nduces organ growth by affecting the Hippo pathway. EMBO J 30: 2325–2335.
10. Lee YJ, Jeong SH, Hong SC, Cho BI, Ha WS, et al. (2013) Prognostic value of CAPZA1 overexpression n gastric cancer. nt J Oncol 42: 1569–1577.
11. senberg G, Aebi U, Pollard TD (1980) An actin-binding protein from Acanthamoeba regulates actin filament polymerization and nteractions. Nature 288: 455–459.
12. Wear MA, Yamashita A, Kim K, Maeda Y, Cooper JA (2003) How capping protein binds the barbed end of the actin filament. Curr Biol 13: 1531–1537.
13. Kim K, McCully ME, Bhattacharya N, Butler B, Sept D, et al. (2007) Structure/function analysis of the nteraction of phosphatidylinositol 4,5-bisphosphate with actin-capping protein: mplications for how capping protein binds the actin filament. J Biol Chem 282: 5871–5879.
14. Wear MA, Cooper JA (2004) Capping protein: new nsights nto mechanism and regulation. Trends Biochem Sci 29: 418–428.
15. Hart MC, Korshunova YO, Cooper JA (1997) Vertebrates have conserved capping protein alpha soforms with specific expression patterns. Cell Motil Cytoskeleton 38: 120–132.
16. Schafer DA, Korshunova YO, Schroer TA, Cooper JA (1994) Differential localization and sequence analysis of capping protein beta-subunit soforms of vertebrates. J Cell Biol 127: 453–465.
17. Hurst S, Howes EA, Coadwell J, Jones R (1998) Expression of a testis-specific putative actin-capping protein associated with the developing acrosome during rat spermiogenesis. Mol Reprod Dev 49: 81–91.
18. Cooper JA, Sept D (2008) New nsights nto mechanism and regulation of actin capping protein. nt Rev Cell Mol Biol 267: 183–206.
19. Yamashita A, Maeda K, Maeda Y (2003) Crystal structure of CapZ: structural basis for actin filament barbed end capping. Embo J 22: 1529–1538.
20. Kim K, Yamashita A, Wear MA, Maeda Y, Cooper JA (2004) Capping protein binding to actin n yeast: biochemical mechanism and physiological relevance. J Cell Biol 164: 567–580.
21. Fernandez BG, Jezowska B, Janody F (3013) Drosophila actin-Capping Protein limits JNK activation by the Src proto-oncogene. Oncogene.
22. Barron-Casella EA, Torres MA, Scherer SW, Heng HH, Tsui LC, et al. (1995) Sequence analysis and chromosomal localization of human Cap Z. Conserved residues within the actin-binding domain may link Cap Z to gelsolin/severin and profilin protein families. J Biol Chem 270: 21472–21479.
23. Schafer DA, Hug C, Cooper JA (1995) nhibition of CapZ during myofibrillogenesis alters assembly of actin filaments. J Cell Biol 128: 61–70.
24. Hart MC, Cooper JA (1999) Vertebrate soforms of actin capping protein beta have distinct functions n vivo. J Cell Biol 147: 1287–1298.
25. Janody F, Treisman JE (2006) Actin capping protein {alpha} maintains vestigial-expressing cells within the Drosophila wing disc epithelium. Development 133: 3349–3357.
26. Amatruda JF, Gattermeir DJ, Karpova TS, Cooper JA (1992) Effects of null mutations and overexpression of capping protein on morphogenesis, actin distribution and polarized secretion n yeast. J Cell Biol 119: 1151–1162.
27. Delalle, Pfleger CM, Buff E, Lueras P, Hariharan K (2005) Mutations n the Drosophila orthologs of the F-actin capping protein alpha- and beta-subunits cause actin accumulation and subsequent retinal degeneration. Genetics 171: 1757–1765.
28. Hopmann R, Cooper JA, Miller KG (1996) Actin organization, bristle morphology, and viability are affected by actin capping protein mutations n Drosophila. J Cell Biol 133: 1293–1305.
29. Frank DJ, Hopmann R, Lenartowska M, Miller KG (2006) Capping Protein and the Arp2/3 Complex Regulate Non-Bundle Actin Filament Assembly to ndirectly Control Actin Bundle Positioning during Drosophila melanogaster Bristle Development. Mol Biol Cell.
30. Gates J, Nowotarski SH, Yin H, Mahaffey JP, Bridges T, et al. (2009) Enabled and Capping protein play mportant roles n shaping cell behavior during Drosophila oogenesis. Dev Biol 333: 90–107.
31. Aragona M, Panciera T, Manfrin A, Giulitti S, Michielin F, et al. (2013) A mechanical checkpoint controls multicellular growth through YAP/TAZ regulation by actin-processing factors. Cell 154: 1047–1059.
32. Rudrapatna VA, Bangi E, Cagan RL (2013) A Jnk-Rho-Actin remodeling positive feedback network directs Src-driven nvasion. Oncogene.
33. Klein T, Arias AM (1998) Different spatial and temporal nteractions between Notch, wingless, and vestigial specify proximal and distal pattern elements of the wing n Drosophila. Dev Biol 194: 196–212.
34. Calleja M, Moreno E, Pelaz S, Morata G (1996) Visualization of gene expression n living adult *Drosophila*. Science 274: 253–255.
35. Wodarz A, Hinz U, Engelbert M, Knust E (1995) Expression of *crumbs* confers apical character on plasma membrane domains of ectodermal epithelia of *Drosophila*. Cell 82: 67–76.
36. Whited JL, Cassell A, Brouillette M, Garrity PA (2004) Dynactin s required to maintain nuclear position within postmitotic Drosophila photoreceptor neurons. Development 131: 4677–4686.
37. Lee JD, Treisman JE (2001) Sightless has homology to transmembrane acyltransferases and s required to generate active Hedgehog protein. Curr Biol 11: 1147–1152.
38. Untergasser A, Cutcutache, Koressaar T, Ye J, Faircloth BC, et al. (2012) Primer3–new capabilities and nterfaces. Nucleic Acids Res 40: e115.
39. Koressaar T, Remm M (2007) Enhancements and modifications of primer design program Primer3. Bioinformatics 23: 1289–1291.
40. Soeno Y, Abe H, Kimura S, Maruyama K, Obinata T (1998) Generation of functional beta-actinin (CapZ) n an E. coli expression system. J Muscle Res Cell Motil 19: 639–646.
41. Schafer DA, Cooper JA (1995) Control of actin assembly at filament ends. Annu Rev Cell Dev Biol 11: 497–518.
42. Fowler VM (1996) Regulation of actin filament length n erythrocytes and striated muscle. Curr Opin Cell Biol 8: 86–96.
43. Johnson RI, Seppa MJ, Cagan RL (2008) The Drosophila CD2AP/CIN85 orthologue Cindr regulates junctions and cytoskeleton dynamics during tissue patterning. J Cell Biol 180: 1191–1204.
44. Lucas EP, Khanal, Gaspar P, Fletcher GC, Polesello C, et al. (2013) The Hippo pathway polarizes the actin cytoskeleton during collective migration of Drosophila border cells. J Cell Biol 201: 875–885.
45. Remmert K, Vullhorst D, Hinssen H (2000) n vitro refolding of heterodimeric CapZ expressed n E. coli as nclusion body protein. Protein Expr Purif 18: 11–19.
46. Ankenbauer T, Kleinschmidt JA, Walsh MJ, Weiner OH, Franke WW (1989) dentification of a widespread nuclear actin binding protein. Nature 342: 822–825.
47. Schafer DA, Mooseker MS, Cooper JA (1992) Localization of capping protein n chicken epithelial cells by mmunofluorescence and biochemical fractionation. J Cell Biol 118: 335–346.
48. Casella JF, Torres MA (1994) nteraction of Cap Z with actin. The NH2-terminal domains of the alpha 1 and beta subunits are not required for actin capping, and alpha 1 beta and alpha 2 beta heterodimers bind differentially to actin. J Biol Chem 269: 6992–6998.
